# Improving Pharmacokinetic-Pharmacodynamic Modeling to Investigate Anti-Infective Chemotherapy with Application to the Current Generation of Antimalarial Drugs

**DOI:** 10.1371/journal.pcbi.1003151

**Published:** 2013-07-18

**Authors:** Katherine Kay, Ian M. Hastings

**Affiliations:** Parasitology Department, Liverpool School of Tropical Medicine, Pembroke Place, Liverpool, United Kingdom; Imperial College London, United Kingdom

## Abstract

Mechanism-based pharmacokinetic-pharmacodynamic (PK/PD) modelling is the standard computational technique for simulating drug treatment of infectious diseases with the potential to enhance our understanding of drug treatment outcomes, drug deployment strategies, and dosing regimens. Standard methodologies assume only a single drug is used, it acts only in its unconverted form, and that oral drugs are instantaneously absorbed across the gut wall to their site of action. For drugs with short half-lives, this absorption period accounts for a significant period of their time in the body. Treatment of infectious diseases often uses combination therapies, so we refined and substantially extended the PK/PD methodologies to incorporate (i) time lags and drug concentration profiles resulting from absorption across the gut wall and, if required, conversion to another active form; (ii) multiple drugs within a treatment combination; (iii) differing modes of action of drugs in the combination: additive, synergistic, antagonistic; (iv) drugs converted to an active metabolite with a similar mode of action. This methodology was applied to a case study of two first-line malaria treatments based on artemisinin combination therapies (ACTs, artemether-lumefantrine and artesunate-mefloquine) where the likelihood of increased artemisinin tolerance/resistance has led to speculation on their continued long-term effectiveness. We note previous estimates of artemisinin kill rate were underestimated by a factor of seven, both the unconverted and converted form of the artemisinins kill parasites and the extended PK/PD methodology produced results consistent with field observations. The simulations predict that a potentially rapid decline in ACT effectiveness is likely to occur as artemisinin resistance spreads, emphasising the importance of containing the spread of artemisinin resistance before it results in widespread drug failure. We found that PK/PD data is generally very poorly reported in the malaria literature, severely reducing its value for subsequent re-application, and we make specific recommendations to improve this situation.

## Author Summary

Pharmacokinetic-pharmacodynamic (PK/PD) models of infectious diseases provide vital insights into the effectiveness of drug treatments (including the optimal dosage level, frequency and duration) by explicitly relating drug concentration after treatment to a pathogen kill rate, and ultimately the models describe whether an infection is likely to be cleared. Furthermore, they can address issues such as poor patient compliance and the spread of drug resistance that are too expensive and/or unethical to determine in the field. Despite their potential, the methodologies used in previous PK/PD models have been based upon the assumptions that only one drug is used in treatment, that the drug is immediately available in its active form at the site of action, and that the parent drug is not further converted to active metabolites. These assumptions severely limit the application of such models. We therefore extend the methodology to remove these assumptions and use this model to investigate two first-line treatments of malaria. The model accurately replicated field data and was then used to predict the impact of increasing drug tolerance and resistance on treatment outcome. We identified key PK/PD data that can, and should, be measured and reported in future field studies to maximise the predictive ability of mathematical models.

## Introduction

Most human infections are currently treatable by drugs. Clinical trials remain the gold standard, empirical approach guiding drug deployment policy and practical issues such as dosing regimes. However *in silico* simulations based on computational predictions of drug treatment outcome have the potential to play a vital ancillary role in designing and guiding these deployment practices. Accurate simulations can rapidly investigate the consequences of putative changes in deployment practices such as changes in regimen (dosage level, frequency and duration of treatment) and can investigate and potentially quantify the threat posed by the evolution of drug resistance. The methodology used to investigate such factors *in silico* is mechanism-based PK/PD modelling, whose basic methodology and range of applications was recently reviewed by Czock and Keller [1]. In essence, this approach incorporates existing PK and PD parameters estimates into differential equations to calculate the decline in drug concentration after treatment, then converts this into a pathogen killing rate to find how pathogen number declines after treatment and whether the infection is eventually cleared. Note the distinction between PK/PD mechanism based modelling (the subject of this manuscript) which uses existing PK estimates to simulate drug treatment, and PK parameter estimation models (usually using non-linear analysis) which are applied to human clinical data to actually produce the PK estimates; a recurring theme of this manuscript is that the former fails to fully utilise all the data produced by the latter and we describe the computational extensions required to achieve this.

PK/PD mechanism-based modelling assumes a single drug is instantaneously present in the patient after treatment (the drug absorption and conversion processes often reported in PK estimation models of human data are ignored) and that pathogens are killed by the drug in its unaltered form [1]. In practice, drug combinations are now mandatory for the treatment of many infections, including the ‘big three’ infective killers HIV, TB and malaria so the single-drug PK/PD methodology needs to be updated to reflect these policies. Many drugs also have short half-lives so the time taken for their absorption (across the gut in the case of oral regimens) may be a significant period relative to half-life and needs to be incorporated into the methodology. Finally, many drugs undergo conversion in the human (often in the liver) to other active forms that also kill the pathogens. This manuscript describes the computational extensions required to update the standard mechanistic-based modelling approach to allow for multiple drugs within a combination, and their absorption/conversion phases. We then illustrate their application to the current batch of first line antimalarial drugs, the artemisinin-based combination therapies (ACTs).

Malaria caused by *Plasmodium falciparum*, is one of the top three infective killers of humans with an estimated 0.75 to 1.5 million deaths per annum [2]. ACTs are now the WHO recommended first-line treatment for uncomplicated malaria [3]. The deployment of these combination therapies was designed to slow or even prevent the evolution of drug resistance which has, historically, been a potent threat to successful malaria treatment; delays in changing policy led to the widespread retention of ineffective drugs and acrimonious accusations of ‘medical malpractice’ aimed at such august institutions as the World Health Organisation [4] and the malaria community must prevent any similar situation arising. However, the policy of deploying ACTs worldwide has lead to increasing levels of artemisinin-tolerance and possibly artemisinin-resistance in *Plasmodium falciparum* being reported on the Cambodia-Thailand border [5], [6], [7], [8], [9] leading to intense speculation about how this will affect the current and future effectiveness of ACTs (e.g. [10], [11]). It is not possible to directly observe the consequences of antimalarial drug resistance until it is too late, so the best approach is to develop the best possible *in silico* models to help guide deployment policies aimed at maintaining long-term effectiveness of these key anti-infective drugs. We therefore apply our updated *in silico* PK/PD modelling methodology to explicitly investigate two front-line ACTs and the public health consequences of increasing tolerance and resistance. Accurate PK/PD modelling has two further important applications. Firstly, it can generate accurate simulations of field data upon which methods of analysis can be developed and refined [12]; the underlying parameters of interest are often unknown in field data but are easily recovered from simulated data enabling the performance of statistical tests to be gauged. Secondly, they can be used to investigate real-life situation that cannot be ethically addressed in the field, an obvious example being poor adherence to a treatment regimen.

## Methods

### Mathematical extensions of the basic model

We use mechanistic PK/PD modelling [1] as previously described in Winter & Hastings [13] with the four key extensions outlined below.

### Pharmacokinetics – incorporating the absorption, conversion and elimination of drugs

**Figure 1:**
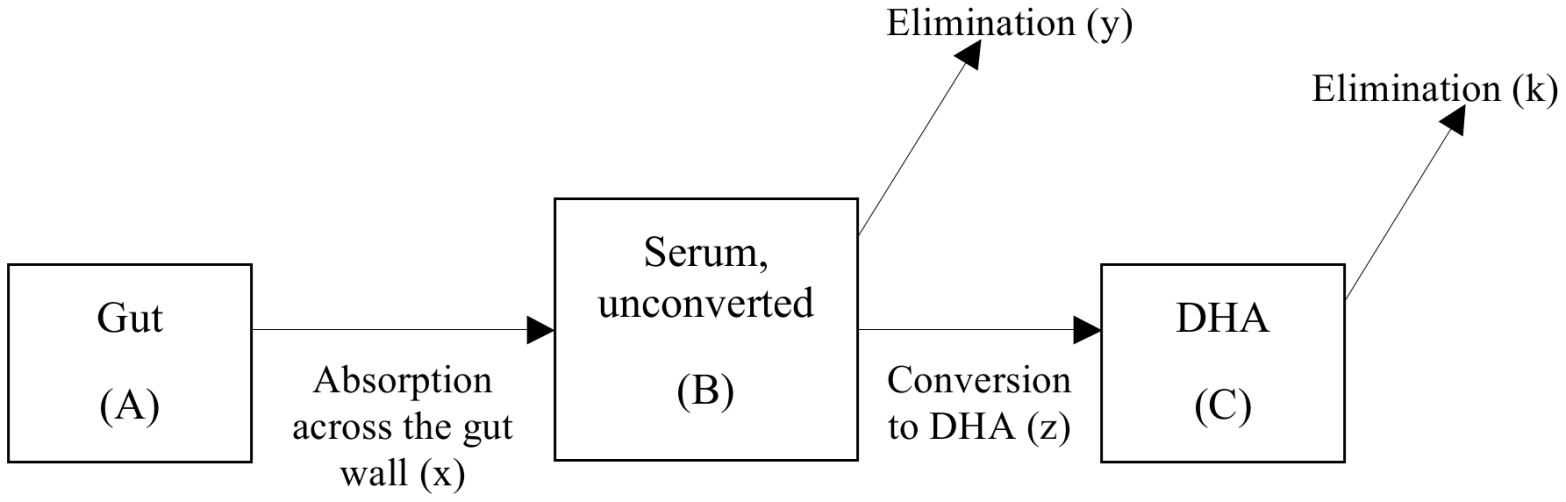
The standard one-compartment pharmacokinetic model. A standard PK one-compartment model allowing for absorption of a drug from the gut (component A) at rate x, into the unconverted form in the serum (component B) where it is eliminated at a rate y and converted into an active form (DHA in this example; component C) at rate z. DHA is then eliminated at rate k.

Standard PK/PD models [1] and their subsequent application to malaria [13], [14], [15], [16], [17] have previously assumed the drugs are instantaneously present in the serum at time *t = *0, are not converted to any other form and decay at a rate *C_t_ = C_0_e^-kt^*, where *C_t_* is the drug concentration at time *t* and *k* is the terminal elimination rate. This assumption is questionable for ACTs as their absorption and subsequent conversion to its active metabolite dihydroartemisinin (DHA) occur over a time period of 1–2 hours, roughly equivalent to their half-life (Figure S1). To address this assumption we track the time course of artemisinin absorption and conversion as illustrated in Figure 1 i.e. absorption across the gut (component *A*) into the serum (component *B*) at rate *x*, its elimination from the body at rate *y* or its conversion to the active metabolite (DHA) (component *C*) at rate *z* and the subsequent elimination of DHA from the body at rate *k*.

The drug-dependent killing function, *f(C)*, was described using the standard Michaelis-Menton equation
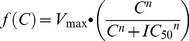
(1)where *C* is the drug concentration (mg/l) which decays over time, *V_max_* is the maximal drug-killing rate (per day), *IC50* is the concentration at which 50% of the maximal killing rate occurs (mg/l) and *n* is the slope of the dose response curve. The problem is therefore to find how *C* varies over time following treatment so that it can be incorporated into Equation 1.

We use a standard one-compartmental model (Figure 1) that appears appropriate for constituents of current ACTs (Text S1), to track the changes in concentration over time. To avoid confusion, we note that “one compartment” is used in the standard PK sense i.e. only one body compartment (in this case, serum) is investigated besides the gut. The change in drug concentration occurring for each component over time (allowing for complications caused by the presence of the drug/metabolite from previous dosages) can be described by three differential equations
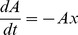
(2)

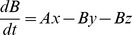
(3)

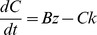
(4)


To find the amount of converted and unconverted drug in the serum at time *t*, Equations 3 and 4 were integrated using laplace transformations [18] (Text S1). Integrating Equation 3 gives
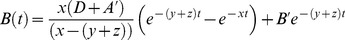
(5)where *B(t)* is the amount (mg) of unconverted drug in the serum at time *t, A′* is the amount (mg) of drug in the gut at the immediate end of the previous time step (time steps correspond to the time between dosages, described in Text S1) i.e. at *t* = 0 (*A′* = 0 if this is the first dose of a multi-dose regimen), *D* is the drug dosage (mg) given and *B′* is the amount (mg) of unconverted drug in the serum at the immediate end of the previous time period i.e. at *t* = 0 (*B′* = 0 if it is the first dose). Inclusion of any drug left over from the previous time step (denoted *A′*, *B′* and *C′*) is essential when including repeat dosages.

Integrating Equation 4 (Text S1) gives
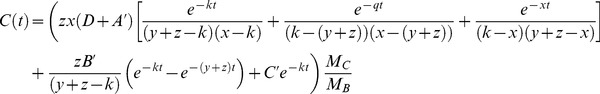
(6)where *C(t)* is the amount of converted drug present in the serum, *k* is the elimination rate of the converted drug, *C′* is the amount (mg) of converted drug in the serum at the immediate end of the previous time step (*C′* = 0 for the first dose) and *M* represents the molecular weight of both the unconverted drug (*M_B_*) and converted drug (*M_C_*). We are tracking drugs in mg so the ratio of the molecular weights of species *B* and *C*, *M_B_* and *M_C_* respectively, are required to account for the changes in molecular weight that occur during conversion.

The drug-dependent killing described in Equation 1 required the amount of drug to be converted to a concentration (mg/l). This was found by dividing the amount of drug by the volume of distribution (l) which is the weight of the patient *W*, multiplied by the volume of distribution *Vd* per kg. The value of *Vd* differs between the drugs so *Vd_B_* and *Vd_c_* represent volumes of distribution for drug forms B and C respectively.

The concentration of component *B* at time *t*, *C_B_(t)*, is therefore
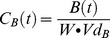
(7)and the concentration of component *C* at time *t*, *C_C_(t)* is
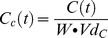
(8)


The use of Laplace transformations in PK is relatively well established [18] so it would be straightforward to extend the calculations for increasing numbers of compartments, drug forms and conversion elimination routes.

The existence of additional compartments in PK estimation models can be taken as an example. To recap, PK/PD mechanism based modelling of malaria requires drug concentrations in the ‘blood’ compartment but all PK estimation models try to include additional compartments where drugs can go; for example a drug may go into a “fat” compartment with fluxes between the blood and fat compartments. PK estimation models decide whether additional compartments are justified by using an information criterion (usually AIC). The problem is that PK estimation modelling is not straightforward and a fair amount of subjective judgement may be required. This subjectivity, combined with different datasets, may result in different analyses of the same drug fitting 1 or 2-compartment models [19]. When using the model it is important that researches maintain consistency in the PK model structures (i.e. assuming one or two compartments). For example, PK parameters derived from a two-compartment model should be incorporated into a PK/PD model that also uses a two-compartment structure. The use of Laplace transforms to incorporate 2 compartmental models is illustrated in the Text S1; users wishing to use a 2 compartmental model can therefore replace equations 5 and 6 obtained above for a one compartment model with Text S1 equations 1.20 and 1.21 obtained from a 2-compartment model.

### Pharmacodynamics – Parasite killing by multiple drugs

The PK/PD modelling now allows for artemisinin absorption and conversion (described above), so the ability to track more than two drug concentrations simultaneously and convert them into a drug-killing rate is crucial. This feature is absent from previous pharmacological models of malaria, which track only a single drug [1] although we previously extended the methodology to track up to two drugs [13]. Existing pharmacological models typically use a standard differential equation [1] to find a mathematical description for the rate of change in total parasite growth and death rates
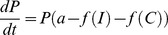
(9)where *P* is the number of parasites in the infection, *t* is time after treatment (days), *a* is the parasite growth rate (per day), *f(C)* represents the drug-dependent rate of parasite killing which depends on the drug concentration *C*, and *f(I)* the killing resulting from the hosts background immunity.

As antimalarial drugs are now typically deployed as combination therapies and as each drug may affect parasites in its unconverted and/or converted forms, predicting the changing numbers of parasites requires an expansion of Equation 9

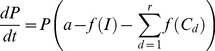
(10)where *r* is the number of drugs, the drug effect *f(C_d_)* is the effect of each drug, *d*. Note that we regard each active entity as a distinct “drug”. For example artemether-lumefantrine (AR-LF) includes three drug forms lumefantrine (LF), artemether (AR) (unconverted) and its active metabolite DHA (dihydroartemisinin). Note that Equation 10 assumes drugs kill independently; this is discussed further below.

Integrating Equation 10 allows us to predict the number of parasites at any time, *t*, after treatment with any number of drugs. This was done by first integrating Equation 9 using the separation-of-variables technique
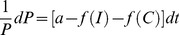
(11)


Integrating both sides of Equation 11 gives
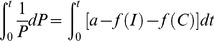
so
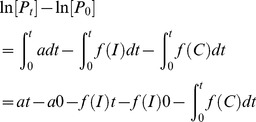



Taking the exponential of both sides (and noting that *a* times *0 = 0*) gives
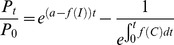
so
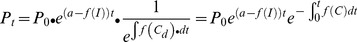
(12)


The problem is now to integrate *f(C).* Assuming there are *r* separate drugs/metabolites with antimalarial activity. In this case, *f(C)* becomes
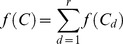
(13)


So for each drug/metabolite *d* we need to calculate its concentration over time *C_d_* using the compartment model Equations (7 and 8) and the substitute *C_d_* into the killing rate equation
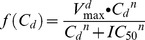
(14)


Note in Equation 14, 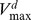 is the maximum drug killing *V_max_* for drug *d*.

Substituting Equation 13 into 12 gives
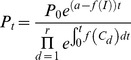
(15)or, equivalently,
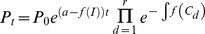
(16)


Note that *C_d_* may be a complicated expression (including Equations 7 and 8) and so 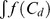 has to be integrated numerically. As before [13], if the predicted parasite number (*P_t_*) falls below 1 we assume the infection has been cleared and the patient cured, immunity is currently ignored (see Winter & Hastings [13] for further discussion).

### Modelling drug killing when two or more drugs are present

These computational extensions to the mechanistic PK/PD modelling allow for the presence of two or more drug forms simultaneously present in the human host, and active against the infection. It therefore becomes necessary to consider and specify how these drug forms interact in their effect against the parasites. There appears to be four main computational choices.

#### Independent modes of action

This is the mode of action explicitly developed above and summarised in Equation 16. Most drug combinations are designed to contain drugs with independent modes of action, so this is a common scenario and would be revealed by drugs having additive action in pharmacodynamic studies [20].

#### Non-independent action

The total drug action may be greater than, or less than, that expected from the sum of the two drugs independently. This is commonly referred to as ‘synergy’ or ‘anatogonism’ but see Chou [20] for a fuller discussion of the dangers inherent in using these terms. It is difficult to even define these terms [20], still less quantify them, so an empirical approach based on data obtained from isobolograms [21] would have to be used to convert drug concentrations into killing.

#### Identical modes of action

This seems plausible if there are different, but structurally similar, forms of same drug. One computational possibility is simply to use the sum of their concentrations in Equation 1 i.e.
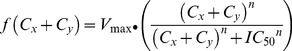
(17)Where x and y are the two forms. Problems arise if *V_max_* or IC50 differ between the two forms. The maximal killing rate may plausibly be the same for each form but it is entirely plausible that structural differences between the forms alter binding of the drugs and hence their IC50 values. It is difficult to compute the joint killing under these circumstances because it is difficult to envisage how to weight the differing IC50 values.

#### Dominant form killing

**Figure 2:**
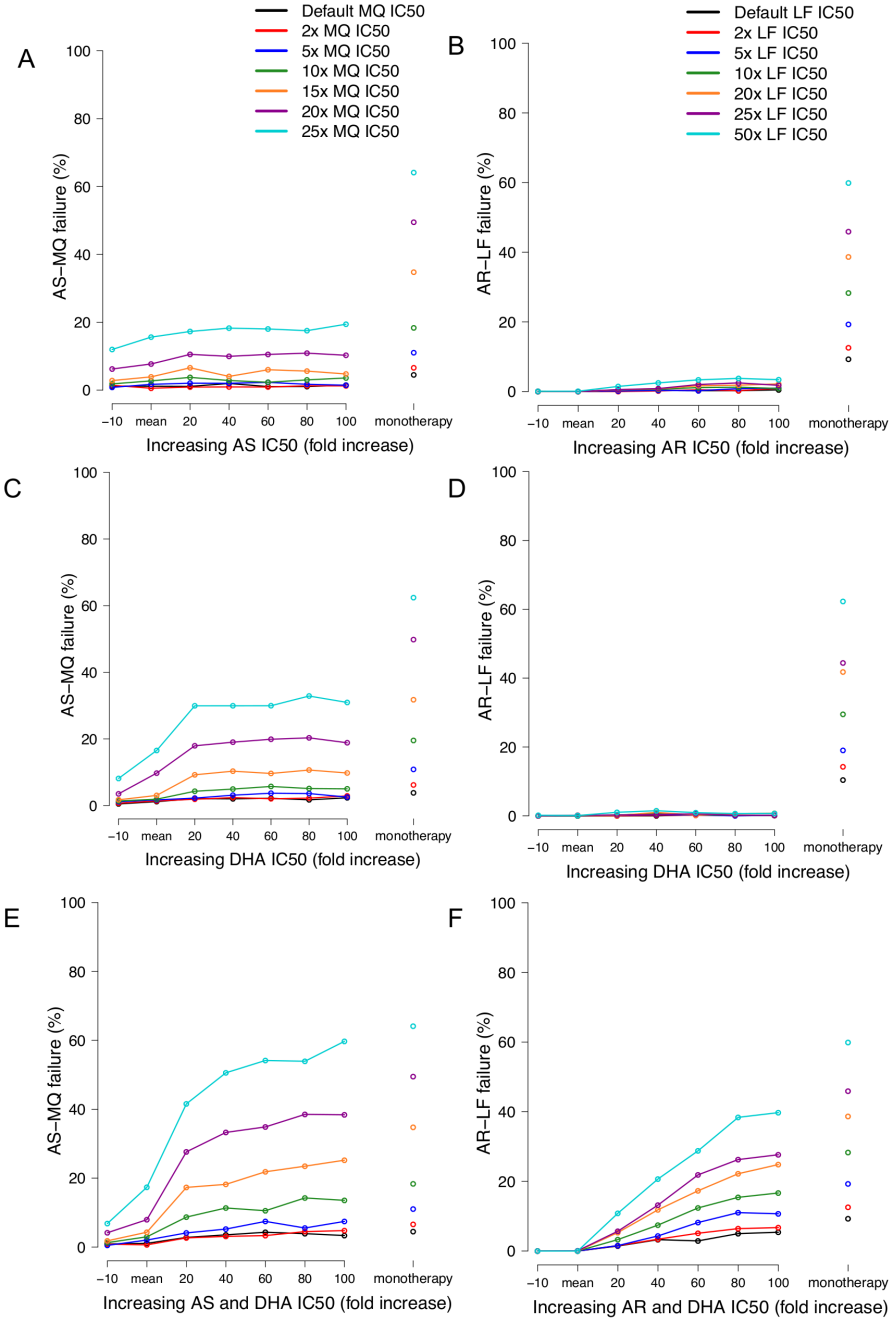
Panels A–F, changes in drug failure rates associated with increasing drug resistance. Changes in failure rates associated with either increasing AS/AR IC50 (panels A–B), increasing DHA IC50 (panels C–D) or simulating increasing both AS/AR and DHA IC50 (panels E–F). Left-hand column includes AS-MQ treatment and the right-hand column AR-LF treatment. Note that failure rates for monotherapies are shown as columns to the immediate right of the x-axis.

This is a computational compromise. The amount of killing of each related drug form over a time period is calculated and the higher killing rate used in the calculations. The underlying premise for this approach is that although related drugs may differ in their IC50s due to minor structural differences (see above), they are likely to have identical effects once present at saturating concentrations (i.e. achieving maximal kill rates according to the Michaelis-Menton Equation 1) because they share the same killing mechanism. If both drugs are present at very low concentrations then kill rate is zero, if one or both are present at very high, saturating, concentrations then killing is equal to their common maximal kill rate. The only problematic situation is where both drugs are present at concentrations resulting in intermediate kill rates. This approach is particularly useful for rapidly eliminated drugs that are essentially either present at full effect or absent so that periods of time with both drug concentrations producing intermediate kill rates can be essentially ignored (see, for example, Figure S2). This is the approach we shall use for artemisinins in the analyses described below. So, for example, when modelling the artemisinins, the drug killing for both forms (i.e. the parent drug and the active metabolite) were calculated during each time step and the drug form with the higher parasite killing was used to update parasite numbers at the end of the time step. As specific examples, either drug killing rate could be used when simulating therapeutic outcome in the patient described by Figure S2A, while artemether kill rate would be used in patients described on Figure 2B, while DHA kill rates would be used in patients whose PK characteristics resulting in them maintaining higher levels of DHA than artemether (example not shown).

### Modelling artemisinin combination therapies

Pharmacological ‘mechanism-based’ modelling [1] has been used previously to investigate key features of antimalarial drug treatment either as monotherapies [14], [15], [16], [17] or with recent emphasis on the current generation of ACTs [13]. We have previously touched upon the potential consequences of increasing artemisinin resistance using standard pharmacokinetic-pharmacodynamic (PK/PD) modelling techniques [13] however, as mentioned in the paper, the model relied heavily on two main assumptions built in to the existing methodology. First, that all drugs are instantaneously absorbed and, if appropriate, converted to their active metabolites. Whilst this may be reasonable for drugs with a long half-life it is not practical for drugs like the artemisinins where absorption and conversion times are almost equal to their short half-lives. The second assumption, that no more than two drugs could be present simultaneously, was reasonable when modelling the ACTs if both drugs were instantaneously absorbed and converted. However, conversion of the artemisinins requires that the artemisinins be modelled as two separate component drugs i.e. the parent drug and the DHA metabolite together with the partner drug and so modelling the ACTs requires a minimum of three drugs be tracked simultaneously. Here we have addressed the methodological challenges of incorporating the absorption and conversion phases of drugs into PK/PD modelling while simultaneously tracking the concentration of more than two drugs, a feature absent in previous pharmacological models [14], [15], [16].

The PK/PD model parameters required to simulate treatment are given in Table S1 and described in the Text S1. The PK extensions for the artemisinins required additional parameters describing the drug absorption rate across the gut, the conversion rate to DHA and the elimination of DHA from the body (Figure 1). These parameters and their associated distributions can be found in Table S1 with details of model calibration and validation included in the Text S1. Variation in model parameters was previously [13] added assuming a coefficient of variation of 30% in all parameters. In reality, some parameters are much more variable [22] while others maybe less so. We now incorporate more appropriate levels of variation into the PK/PD parameters using drug specific distributions thus making results more compelling for specific ACTs. To validate the model's predictive ability, the maximum serum concentration (Cmax) and time to achieve Cmax (Tmax) were compared to field data (Text S1).

The methodology described above now allows for the action of both the unconverted and converted forms of the artemisinins. However, given that they have similar modes of action their effect on parasite numbers is unlikely to be additive (as is assumed in Equation 11). As such, the drug effect, *f(C)*, for each of the artemisinin forms was calculated each time-step but only the dominant form (i.e. parent drug or active metabolite) with the greater drug killing effect was used to compute the number of parasites in the next time step. Activity, and hence killing, of artemisinins and the partner drug were assumed to be independent.

A major change was made to the artemisinin maximal drug kill rate (*V_max_*). Previous estimates of the *V_max_* [13], [23], [24] have been based upon the assumption that drug killing is maximal immediately after treatment and remains so for 48 hours after treatment. This is quantified by the parasite reduction ratio (PRR); a ratio of the number of parasites at time of treatment scaled by their number 48 hours after treatment. So, assuming the decline in parasitaemia is first order, the parasite count (*P_t_*) at any given time (*t*) is given by
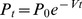
(18)where *P_0_* is the number of parasites present at the start of treatment.

This appears to be reasonable for drugs given at relatively high doses with a long half-life because the maximal killing will extend over the 48 hours after treatment. However, it is unrealistic for the artemisinins whose short half-lives mean parasites are typically only exposed to high concentrations of artemisinins during the first 6–8 hours following treatment (Figures S1 and S2). The steady decline in parasite numbers after this period presumably reflects dead or dying parasites being cleared by host mechanisms. PK/PD modelling of drug effect assumes deaths only occur in the presence of the drug (i.e. 6–8 hours post-treatment) hence the need for this increased kill rate. So, given PRR = P_0_/P_t_ [23] (where P_t_ is usually assumed to be 48 hours), the relationship between PRR and parasite killing rate *V_max_* is
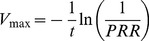
(19)When *t* is assumed to be 48 hours and PRR is 10^4^ then the maximal artemisinin drug kill rate (*V_max_*) is 4.6 as used previously by ourselves and others [13], [23]; we now consider that value inappropriate because a 6 hour burst acting at a kill rate of 4.6 would achieve a PRR of well below 10^4^ . Consequently, we assume artemisinin maximal drug killing occurs only during the 6 hours when the drugs are actually present at therapeutic concentration (Figures S1 and S2), so achieving a PRR of 1000 (White [23] gives a range of 10^3^ to 10^5^ for the artemisinins) requires *V_max_* to be 27.6. Note, if the maximal drug killing is assumed to occur over 8 hours and the PRR is assumed to be 10,000 (within the range reported in White [23]) *V_max_* again equals 27.6. Consequently our artemisinin maximum killing rate is approximately 7-fold higher than in previous simulations.

Two treatment combinations were investigated, artesunate-mefloquine (AS-MQ) and AR-LF, both are highly effective ACTs currently used to treat malaria. Variation in how humans metabolise the drug and parasite drug sensitivity was added to the model parameters (Table S1) using parameter specific estimates of co-efficient of variation, CV. The technical details regarding parameter variability are included in the Text S1.

The extended pharmacokinetic-pharmacodynamic (PK/PD) model can then be implemented to address a critical feature of current ACT deployment: how is the observed increase in artemisinin tolerance likely to affect the long-term effectiveness of ACTs? The crucial operational question is whether there is likely to be a sudden catastrophic decrease in ACT effectiveness, a gradual decline or, a best case scenario, a margin of safety such that we can have relatively large increases in artemisinin tolerance/resistance before ACT failures start to increase?

The partner drugs, LF and MQ, are currently largely effective monotherapies if administered correctly (although MQ in south east Asia may be problematic) so increasing artemisinin resistance would, by definition, have little or no impact on therapeutic outcome. To avoid this trivial case, we investigated how increasing levels of artemisinin resistance impacted treatment failure rates if resistance to the partner drug was already present or spreading. When modelling MQ treatments the MQ IC50 values were either 1-, 2-, 5-, 10-, 15-, 20- or 25-fold greater than the current default value (Table S1) and when modelling LF treatments LF IC50 values were either 1-, 2-, 5-, 10-, 20-, 25- or 50-fold greater than the current default value (Table S1). Resistance to artemisinins was investigated in two ways. First by increasing the IC50 of the AS, AR or DHA (the active metabolite) independently and then by assuming the IC50s of the parent species and DHA were completely correlated i.e. the IC50s were increased simultaneously by the same amount. This was necessary because it is not clear whether parasites will evolve resistance independently to the artemisinin entities or whether there will be substantial cross-resistance to different entities (see later discussion) The IC50 range of both artemisinin forms included one value 10-fold smaller than the mean and values 1-, 20-, 40-, 80- or 100-fold greater than the mean.

Details of implementation are in the Text S1. For each of the 10,000 patients simulated the model recorded whether an infection (with one clone) was cleared and, if so, the parasite clearance time (PCT; defined as the time taken for an infection to fall below the limit of microscopic detection, which was assumed to be 10^8^). This was done first for the partner drugs without the artemisinin component, i.e. as monotherapies, to give a baseline failure rate. Then, by comparing the results of the monotherapy with those of the ACTs we were able to quantify the ability of the artemisinin component to reduce failure rates and PCTs.

## Results

The artemisinin drug concentration profiles of the model are consistent with those measured in the field (discussed in Text S1 and Figure S1). Analysis of both ACTs showed that adding an artemisinin to a partner drug reduced failure rates below that of the monotherapy regardless of the initial levels of partner drug resistance, the latter being achieved through varying the partner drug IC50 value (Figure 2); the only exception was the trivial case when partner drugs were fully effective as monotherapies. For AS-MQ, the exact proportion of failures prevented by the artemisinin component was dependent on the initial level of resistance to the partner drug. Regardless of whether the IC50s of the artemisinins were correlated, adding an artemisinin at its default IC50 value to a partner drug reduced failure rates by between 70 and 90%. This is a relative reduction, for example, a 50% reduction is equivalent to fall in failure rates from 40% to 20% or from 12% to 6% (Figure 2, panels A, C and E). This is consistent with field observations that adding AS to MQ reduced the absolute risk of failing treatment but did not result in a fully effective ACT [25]; this has also been observed for other failing monotherapies not modelled here (chloroquine, amodiaquine, sulfadoxine-pyrimethamine) [25]. The results also show that the addition of AR to LF monotherapies reduced failure rates to zero when modelling the mean parameter values (Figure 2, panels B, D and F).

Figure 2 shows the failure rates of the ACTs when the IC50s of the two artemisinin drug forms were either varied independently (Figure 2, panels A to D) or varied simultaneously (Figure 2, panels E and F). When the IC50s of the artemisinin drug forms were varied independently increasing the IC50 of either had very little effect in the failure rates (Figure 2, panels A, B, C and D). This was particularly clear for AR-LF treatments where increasing either AR or DHA IC50 caused no measurable increase in drug failure rates (Figure 2, panels B and D). This occurs because resistance to one form is compensated by continued sensitivity to the other form because both forms are potentially capable of high rates of parasite killing (Figure S2). Increasing AS IC50 alone also had little effect on the AS-MQ failure rates (Figure 2, panel A), again highlighting the importance of its active metabolite on parasite survival. When DHA IC50 was increased by 20-fold in AS-MQ treatment (Figure 2, panel C), treatment failures increased by 25 to 65% (relative increase) depending on the level of resistance to the partner drug. This is the only time increasing either the artemisinin drug forms alone affected treatment outcome and further DHA IC50 increases (above 20-fold) had little further effect on treatment outcome (Figure 2, panel C). Failure rates to AS-MQ assuming the artemisinin drug forms were uncorrelated (Figure 2, panels A and C) remained lower than those seen when assuming they were correlated (Figure 2, panel E) thus implying both artemisinin drug forms are still playing an active role in parasite killing.

Further DHA IC50 increases above 20-fold had no discernable effect on treatment outcome and failure rates remained lower than those seen when the IC50's were correlated thus implying that while not as potent as AR and DHA it still plays an active role in parasite killing. For both ACTs, increases in failure rate as a result of increasing artemisinin resistance were much larger if the IC50s of the artemisinin drug forms were simultaneously increased. Rapid loss of protection was most noticeable for AS-MQ with small IC50 increases (20 and 40-fold), well within the range of natural variation [22], increasing failure rates by 65–70% (Figure 2, panel E). Loss of protection was more gradual following AR-LF treatments (Figure 2, panel F) but both ACTs showed failure approaching those of the of the monotherapies as artemisinin IC50s increased to 100-fold greater than the mean.

**Figure 3:**
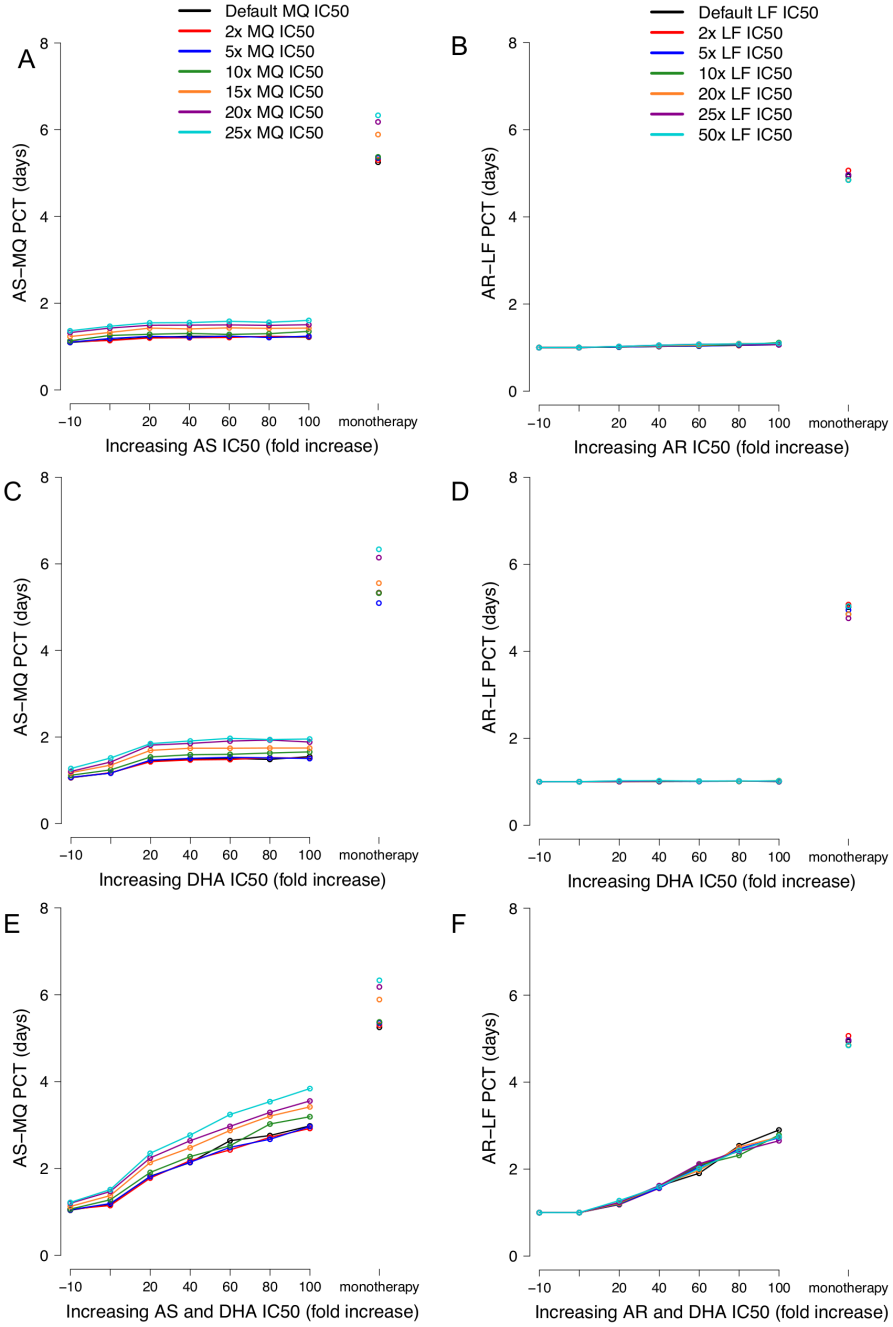
Panels A–F, changes in parasite clearance times associated with increasing drug resistance. Changes in parasite clearance times (PCT) associated with either increasing AS/AR IC50 (panels A–B), increasing DHA IC50 (panels C–D) or simultaneously increasing both AS/AR and DHA IC50 (panels E–F). Left-hand column includes AS-MQ treatment and the right-hand column AR-LF treatment. Note that PCTs for monotherapies are shown as columns to the immediate right of the x-axis.

The PCT appears to be determined predominantly by the level of resistance to the artemisinin component with the initial level of partner drug resistance being relatively unimportant (Figure 3). This was particularly evident following AR-LF treatment where increasing the IC50 of LF had no discernable effect on PCT (Figure 3, panels B and D) while increasing MQ resistance only caused the PCT to vary by up to one day (Figure 3, panels A and C). When the IC50s of the two artemisinin species were increased simultaneously, the addition of artemisinin to the monotherapy reduced PCTs by approximately 2 to 3 days for both ACTs. As seen with the treatment failures (Figure 2), increasing the IC50 of AS/AR or DHA independently had little/no effect on PCT (Figure 3, panels A to D) and PCT did not approach that of the monotherapy because the other artemisinin species retained its effectiveness. When the IC50s were increased simultaneously both artemisinin species lost their effectiveness (Figure 3, panels E and F) while the PCT increased almost linearly with increasing artemisinin resistance and approached the PCTs seen with monotherapies (Figure 3, panels E and F).

## Discussion

The extended PK/PD mechanism based modelling was applied to ACTs and produced results and predictions consistent with field data on failure rates [25] and increasing PCT associated with resistance. The main operational concern surrounding the evolution of artemisinin resistance is that it will lead to clinical failure in patients treated with ACTs [26]. Obviously, if the partner drug is effective as a monotherapy, then the presence or absence of artemisinin resistance has no clinical effect. Problems arise as resistance spreads to the partner drugs, a process slowed by the addition of an artemisinin [27]. The results clearly show that adding AS to a failing drug (MQ) reduced the treatment failure rates by up to 90% (relative reduction) but did not result in a fully effective ACT (Figure 2, panel E). This observations is in line with the findings of the International Artemisinin Study Group who performed a meta-analysis of individual patients from 16 randomised trials (n = 5948) studying the effect of adding AS to either CQ, AQ, SP or MQ [25]. While the total population failure rates were reduced by 42–65% when averaged across all drug regimens, the addition of AS to MQ monotherapy reduced failure rates by approximately 90–95% [25]. The results for AR-LF show that the addition of AR with default IC50 values was sufficient to save a failing LF monotherapy by reducing failure rates to <1% for all levels of partner drug resistance regardless of whether the IC50s of the AR and DHA are increased simultaneously or independently (Figure 2, panels B, D and F). However, this observation was much more difficult to validate than those of AS-MQ as there is almost no published data on the *in vivo* efficacy of LF monotherapy and so it is impossible to quantify the proportion of failures averted specifically by the addition of AR. We also note that for both ACTs, only when the IC50s were correlated did increasing the IC50 eventually lead to failure rates approximately equal to those of the monotherapy therefore removing any benefit afforded to the partner drug by the artemisinin. These occurred after 50–100 fold increases in artemisinin IC50 which is large, but around the same magnitude as the natural variation observed in field isolates [22]. The key question is whether the IC50s are correlated; field data suggest they are (Text S1).

Increasing PCTs are currently being observed in the field [7], [26], [28], [29], [30]; Dondorp *et al*. [31] for example, show that parasites resistant or tolerant to artemisinins take 3 or 4 days to parasites as compared with less than 2 days for artemisinin sensitive parasites; this pattern was also apparent in the results presented here (Figure 3). The simulated results showed the initial level of resistance to the partner drug had very little effect on the PCT and whilst this may seem strange it can be explained relatively easily. While the partner drug is undeniably important when determining the treatment outcome (i.e. success or failure), the PCT is determined almost solely by the short-lived but fast-acting artemisinin component, which causes a rapid decline in parasite numbers but is not present long enough to completely clear the parasite load [13]. As with dug failure rates, PCT only approached those of the monotherapies when the IC50s were increased simultaneously again consistent with field data that the IC50s are correlated (Text S1). For both ACTs, PCT began to increase after relatively small increases in artemisinin IC50 of 20- to 40-fold (within the range of natural variation [22]).

The results shown on Figure 2 illustrate an important factor not generally recognised when considering how resistance may arise to artemisinins and other drugs whose converted and unconverted forms are both active: if resistance arises to only one form, then the other form may retain sufficient activity to compensate. This is well illustrated by AR-LF in Figure 2 where increasing resistance to either AR alone (Figure 2B) or DHA alone (Figure 2D) has virtually no impact on failure rates which only start to escalate if resistance occurs simultaneously to both forms (Figure 2F). It is therefore essential to consider whether mutations that encode resistance to one form are likely to simultaneously encode cross-resistance to the other form (so that IC50s are correlated), or whether the mutations are specific to individual drug forms (in which case IC50s are uncorrelated). When considering the likelihood of cross-resistance, it is important to realise that cross-resistance and mode of drug action are related, but distinct entities. Drugs with identical modes of action may show complete cross-resistance if mutations occur at their site of action which prevents both/all forms of the drug from binding therefore blocking their activity. Alternately, resistance may emerge through mutations that alter the drugs' ability to reach or accumulate at their site of action. Malaria is often characterised by the latter where mutations in membrane transporters, notably mdr and crt, are implicated in resistance to a range of antimalarial drugs [32]. These transporters depend more on the chemical scaffold (charge and structure) of the drug than its active site so it is not *a priori* certain that cross-resistance will inevitable occur between a parent drug and its active metabolite. A lack of cross resistance would be hugely beneficial as it means parasites would have to evolve resistance to both forms of the drug but, unfortunately, our simulations suggest a model of complete cross resistance provides the best fit to the malaria observations that IC50s are likely to be correlated (discussed further in SI).

Drug IC50 values are estimated either from parasites taken from a patient's primary infection or from laboratory isolates. The IC50 values of the artemisinins and their active metabolite DHA vary widely in the literature and their reported values appear to be highly dependent on the source from which they were estimated. For example, Brockman *et al.* [33] show the mean IC50 of AR was approximately 4-fold higher than DHA (4.83 and 1.22 respectively) when measured in patients from Thailand but were approximately equal (3.4 and 3.6 in 1996 and 3.1 and 4.0 in 1998 respectively) when measured in K1 laboratory isolates. The 4-fold lower DHA IC50 measured in patients may result in a higher level of effectiveness of DHA in their patient population. What is not generally realised is that both artemisinin components are potentially important in determining treatment outcome; for example Saralamba *et al*. [34] simply stated that in their patients “the total drug exposure of AS was <10% that of DHA” and so choose to ignore the parasiticidal effect of AS. This may be true *on average*, but there is huge variation in how patients metabolise different forms of the drug so it entirely plausible that some patients will slowly convert artesunate but rapidly clear DHA, in which case the former would have the larger killing effect. In particular, changing IC50 simply translates into how long the drug is killing at near-maximal rates in the few hours following treatment (Figure S2). Importantly, this means that artemisinin therapy given as artesunate or artemether has an inherent therapeutic safety margin: If one component of the artemisinin is metabolised quickly or has a particularly high volume of distribution, there is still a second active component present within the patient that is likely to retain therapeutic effectiveness.

Increasing tolerance/resistance to artemisinins was modelled using the standard assumption that it will arise through increased IC50 values. Artemisinin resistance may be atypical in this respect as it appears to manifest through increased clearance times of parasites following treatment with unchanged IC50, possible due to the drug(s) having activity against a more restricted range of stages in the malaria cell cycle (see below). The mechanistic approach assumes instantaneous killing of parasites irrespective of their stage, so deceased activity against some stages would be manifested as decreased drug maximal killing rate (*V_max_* in Equation 1) in the methodology; interesting this parameter was found to be a far more potent determinant of resistance than the IC50 [13]. It would be possible to re-run the above simulations altering *V_max_* rather than IC50 but we chose to use the more conventional approach in the first instance as we consider this primarily a computational paper; we shall explore this approach in future studies applying the methodology more specifically to malaria.

Malaria differs from many other pathogens in having a distinct 48 hour intracellular cycle that essentially consists of invasion of red blood cells (RBC), digestion of host haemoglobin, parasite multiplication within the RBC, cell rupture and re-invasion of new RBCs. Drugs consequently have different stage specificity profiles depending on what metabolic processes are occurring in each stage (for example, many drugs target haemoglobin digestion so are primarily active against parasites in this stage of their cycle). Our analyses ignored these drug stage-specificities. It would however be easy to re-compute the dynamics using one hour time steps and using a 48 hour array to move parasites through the 48 hour development cycle as done previously [35], [36], [37]. We chose not to do so for two main reasons. Firstly, stage specificity requires that PD parameters be specified for each stage and that the initial distribution of parasite stages in the infection be specified. Secondly, and more importantly in our opinion, is that the PK/PD computations assume instantaneous killing of parasites depending on current drug concentration whereas, in reality, there is a delay in killing. The delayed killing can be incorporated into the methodology by postulating a hypothetical ‘metabolite’ whose production or elimination is disrupted by the drug, and that parasite death occurs as a function of metabolite level; the time taken for metabolite levels to reach ‘lethal’ levels introduces a time-lag into the killing [38], [39]. This is an elegant way of incorporating a delay but it requires further parameterisation of the metabolite's production and elimination, specification of a killing rate as a function of metabolite level, and calibration against field data. Patel and colleagues [38] estimated the delay in artemisinin killing as around 5 hours. A recent study attempted to simulate ACT dynamics using a stage structured approach and concluded that it did not match well field data [36]; we are unsurprised because the short-term dynamics will be critically dependent on stage-specific PD parameterisation and no time lag was built into the model. Hence, our approach was to ignore short-term dynamics and run the enhanced PK/PD methodology, ignoring stage specify and delayed drug action [40]; the objective was to simulate the fate of the infection over the longer term rather than the dynamics immediately post-treatment. Consistency of our results with field and clinical observations suggest this is a robust approach but it is important to recognise the alternative modelling approaches can be designed, and that our enhanced PK/PD methodology can easily form the basis for an improved stage-specific model run in 1-hour time steps.

The rationale behind this paper is that combining good quality field and clinical data into a sophisticated PK/PD model should allow a thorough investigation of ACT effectiveness in the context of increasing artemisinin tolerance/resistance. It therefore provides a methodological framework for clinical pharmacologists to interpret their results. However the predictive power of mathematical modelling is governed by the crucial step of model calibration and the availability of comprehensive, good quality PK/PD data in the literature is surprisingly scarce (Supporting Information, part 2). This has the potential to limit the usefulness of models as predictive tools. Given the amount of effort and resources required to conduct PK/PD studies and that their explicit aim is usually to improve human therapy, it seems appropriate to consider how best to report such studies for maximum impact. We therefore make three specific suggestions that authors may consider to maximise their studies' chance of influencing policy choice. Firstly, all available population PK/PD data, including those required purely for intermediate calculations should be reported. For example, terminal elimination rates are invariably reported but parameters required in their calculation, for example volumes of distributions (often confounded with bioavailability) are often omitted [41]. We are uncomfortable with the rationale underlying the common assertion that DHA is the main active species during artemisinin treatment (see above and Figure S2); we would therefore recommend that PK parameters for parent species such as artesunate and artemether also be measured and reported. Secondly, the nature and extent of natural variation in the parameters are vitally important and can result in some patients developing low drug concentrations possibly leading to therapeutic failures or high concentrations potentially leading to toxicity. The distributions (normal, log-normal, etc) with their associated coefficients of variations (CV) are therefore almost equally important as their mean values. For example, many authors cite CV estimates larger than the mean, which obviously indicates a non-normal distribution: such data are much more useful if accompanied by their distributions (herein we were forced to assume they were log-normal). Finally, there are wide variations in reported mean values between studies; these are generally ascribed to sampling different populations or age groups but a more critical appraisal in terms of any impact of different methods of analysis would also be helpful. An excellent example is that of Tan *et al*. [42] who, after describing the population PK of AS and DHA in healthy patients, compare their results with those of other AS and DHA PK studies and provide a detailed discussion explaining how and why the results may differ.

We would emphasise that our choice of specific studies to parameterise the simulation should not be regarded as prescriptive or judgemental; as described above, the choice was often problematic. Few, if any, PK/PD studies produce all the parameters required to evaluate their impact on therapeutic outcome. PK studies often focus on a single drug in a combination and lack local estimates of parasite drug sensitivity, while PD studies generally lack accompanying PK estimates. Consequently, we have focused on developing a methodology that individual researchers can calibrate as they wish; we provide a mechanism by which their results can be integrated with the results of other studies to gauge their implications for drug effectiveness.

Despite the caveats mentioned above, our results and implications are clear. The kill rate of both artemisinin forms appears to be important in determining treatment outcome and their IC50's are likely to be correlated. AS-MQ is more sensitive to increases in artemisinin drug resistance than AR-LF with the number of failures increasing quickly with relatively small increases in AS and DHA IC50s. Both ACTs show increasing PCT associated with increasing artemisinin IC50, an observation already seen in the field [5], [6], [7], [8], [9]. Our results suggest this is indicative of a rapid loss of protection provided by the artemisinins against the partner drug(s). If, or when, resistance against the partner drug starts to increase, most plausibly driven by mismatched half-lives [43], [44], [45], then a rapid reduction in ACT clinical effectiveness is likely to occur. We conclude that policies designed to isolate and minimise the spread of artemisinin resistance are to be greatly encouraged [26].

## Supporting Information

Figure S1 **Panels A–C, the simulated PK profiles of the artemisinins and the relationship between drug concentration and drug killing rate.** The simulated PK profiles of the artemisinin forms given as the parent drug and subsequently converted to DHA. Given as (A) artesunate or (B) artemether; generated using the model shown in Figure 1 mathematical derivation described herein and using the parameters of Table S1. The timescale and concentrations match well with those observed *in vivo* (see, for example, [47], [48], [49]). Note that DHA is the major component when dosing with artesunate, but the minor component when dosing with artemether. Panel C shows the relationship between drug concentration and killing rate as described by the Michaelis-Menton Equation 1 in the main text. All Figures were produced using the default parameter values given in Table S1.
(TIF)

Figure S2 **Panels A–B, the simulated parasite kill curves of the artemisinins.** The simulated parasite kill curves of the parent artemisinin drug forms (artesunate and artemether) and their active metabolite DHA. Treatment with (A) artesunate and (B) artemether. Curves generated using the mathematical derivation described herein and using the parameters of Table S1.
(TIF)

Figure S3 **Panels A–B, changes in drug failure rates associated with increasing drug resistance when parameters are varied by 30%.** Change in failure rates associated with increasing AS/AR and DHA IC50 when the coefficient of variation in all parameters is always 30% (A) AS-MQ treatment and (B) AR-LF treatment.
(TIF)

Figure S4 **Panels A–F, changes in drug failure rates associated with increasing drug resistance when assuming independent action of the artemisinin components.** Change in failure rates associated with either increasing AS/AR IC50 (panels A–B), increasing DHA IC50 (panels C–D) or increasing AS/AR and DHA IC50 (panels E–F), AS-MQ treatment (left column) and AR-LF treatment (right column) assuming independent action of the artemisinin components. Note that failure rates for monotherapies are shown as columns to the immediate right of the x-axis.
(TIF)

Figure S5 **Panels A–F, changes in parasite clearance times associated with increasing drug resistance when assuming independent action of the artemisinin components.** Change in parasite clearance times (PCT) associated with either increasing AS/AR IC50 (panels A–B), increasing DHA IC50 (panels C–D) or increasing AS/AR and DHA IC50 (panels E–F), AS-MQ treatment (left column) and AR-LF treatment (right column) assuming independent action of the artemisinin components. Note that PCTs for monotherapies are shown as columns to the immediate right of the x-axis.
(TIF)

Figure S6 **The standard two-compartment pharmacokinetic model.** A standard PK two-compartment model allowing for drug absorption from the gut (component A) to the central compartment (component B) at a rate x. The drug is either eliminated from the body at a rate k or exchanged with a peripheral compartment (component C)), the drug leaves the central compartment at a rate y and returns at a rate z.
(TIF)

Table S1 **Mean antimalarial drug parameters and their associated distributions.** Mean antimalarial drug parameters for artesunate-mefloquine and artemether-lumefantrine combination therapies. The amount of variation (i.e. CV) is given in square brackets.
(DOCX)

Table S2 **Correlations between the IC50s of five antimalarial drugs.** Data describing the half-maximal inhibitory concentration (IC50) of 5 different antimalarials measured in 7 different *P.falciparum* strains by Delves *et al.* [46], was used to determine whether the IC50s of the artemisinins are correlated.
(DOCX)

Text S1 **Pharmacokinetic model extensions, model calibration and implementation.** Supporting information.
(DOCX)
