# Supplementary figures and images for: Improving Pharmacokinetic-Pharmacodynamic Modeling to Investigate Anti-Infective Chemotherapy with Application to the Current Generation of Antimalarial Drugs
Source: PLoS Comput Biol. 2013 Jul 18;9(7):e1003151. doi: 10.1371/journal.pcbi.1003151 (PMC3715401; doi:10.1371/journal.pcbi.1003151)

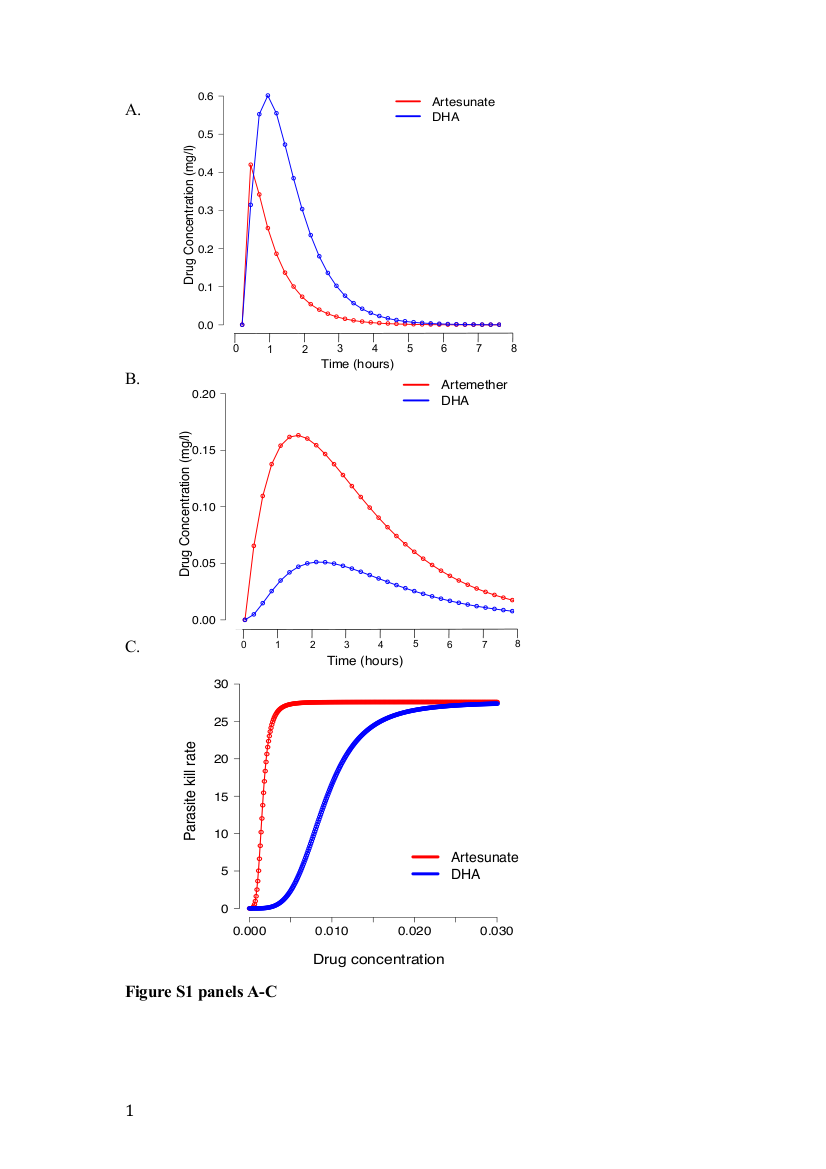

Supplement: Figure S1 — Panels A–C, the simulated PK profiles of the artemisinins and the relationship between drug concentration and drug killing rate. The simulated PK profiles of the artemisinin forms given as the parent drug and subsequently converted to DHA. Given as (A) artesunate or (B) artemether; generated using the model shown in Figure 1 mathematical derivation described herein and using the parameters of Table S1. The timescale and concentrations match well with those observed in vivo (see, for example, [47], [48], [49]). Note that DHA is the major component when dosing with artesunate, but the minor component when dosing with artemether. Panel C shows the relationship between drug concentration and killing rate as described by the Michaelis-Menton Equation 1 in the main text. All Figures were produced using the default parameter values given in Table S1. (TIF) [file pcbi.1003151.s001.tif]

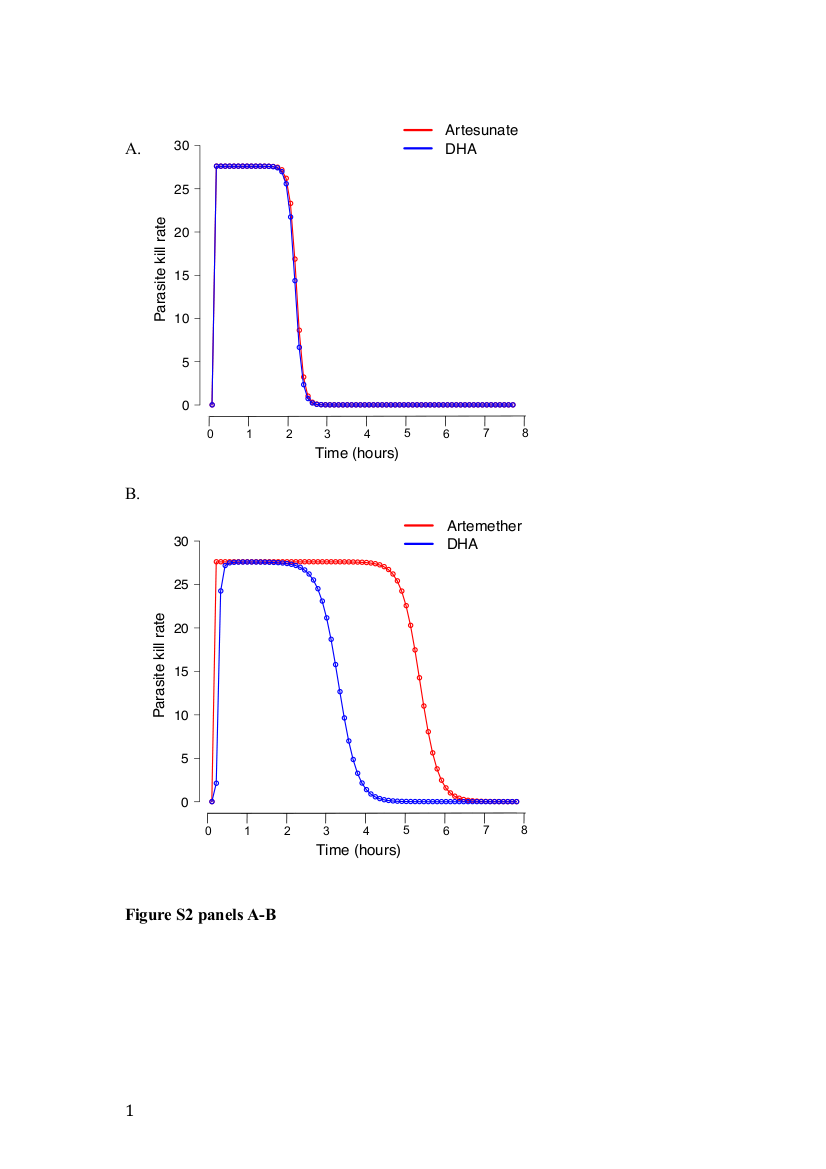

Supplement: Figure S2 — Panels A–B, the simulated parasite kill curves of the artemisinins. The simulated parasite kill curves of the parent artemisinin drug forms (artesunate and artemether) and their active metabolite DHA. Treatment with (A) artesunate and (B) artemether. Curves generated using the mathematical derivation described herein and using the parameters of Table S1. (TIF) [file pcbi.1003151.s002.tif]

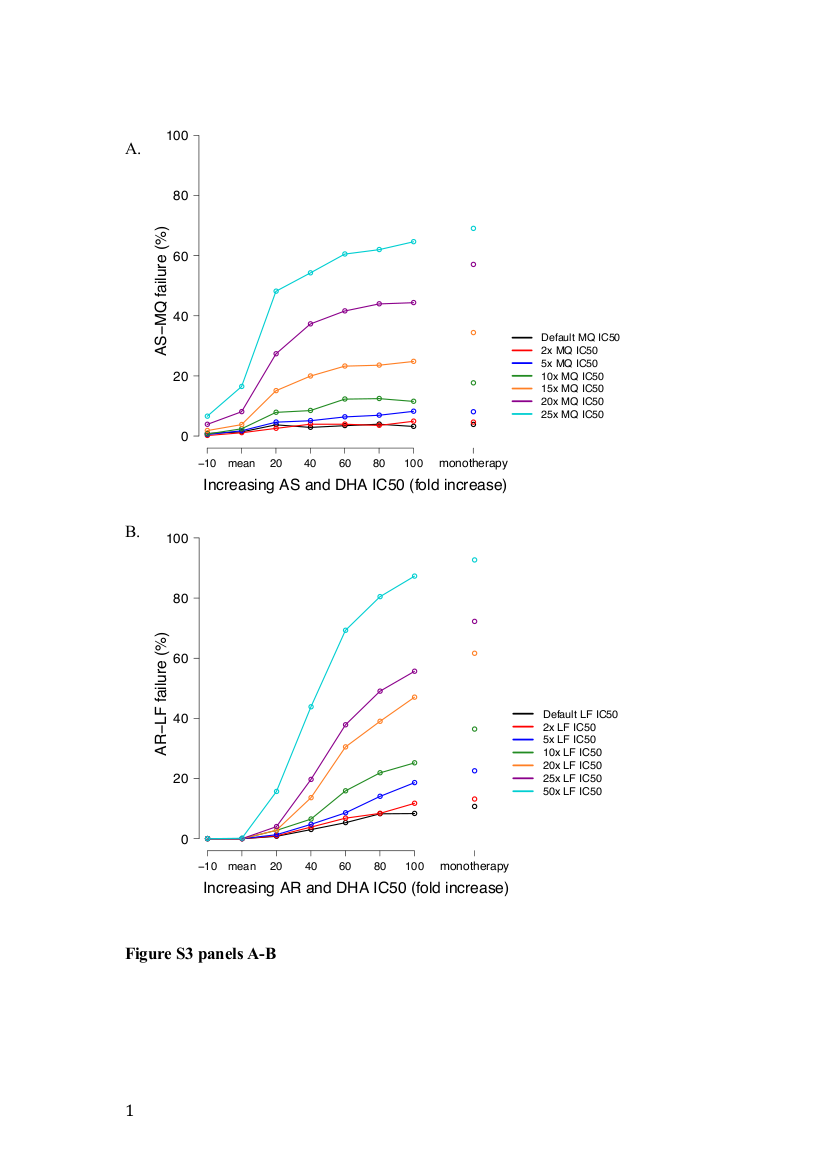

Supplement: Figure S3 — Panels A–B, changes in drug failure rates associated with increasing drug resistance when parameters are varied by 30%. Change in failure rates associated with increasing AS/AR and DHA IC50 when the coefficient of variation in all parameters is always 30% (A) AS-MQ treatment and (B) AR-LF treatment. (TIF) [file pcbi.1003151.s003.tif]

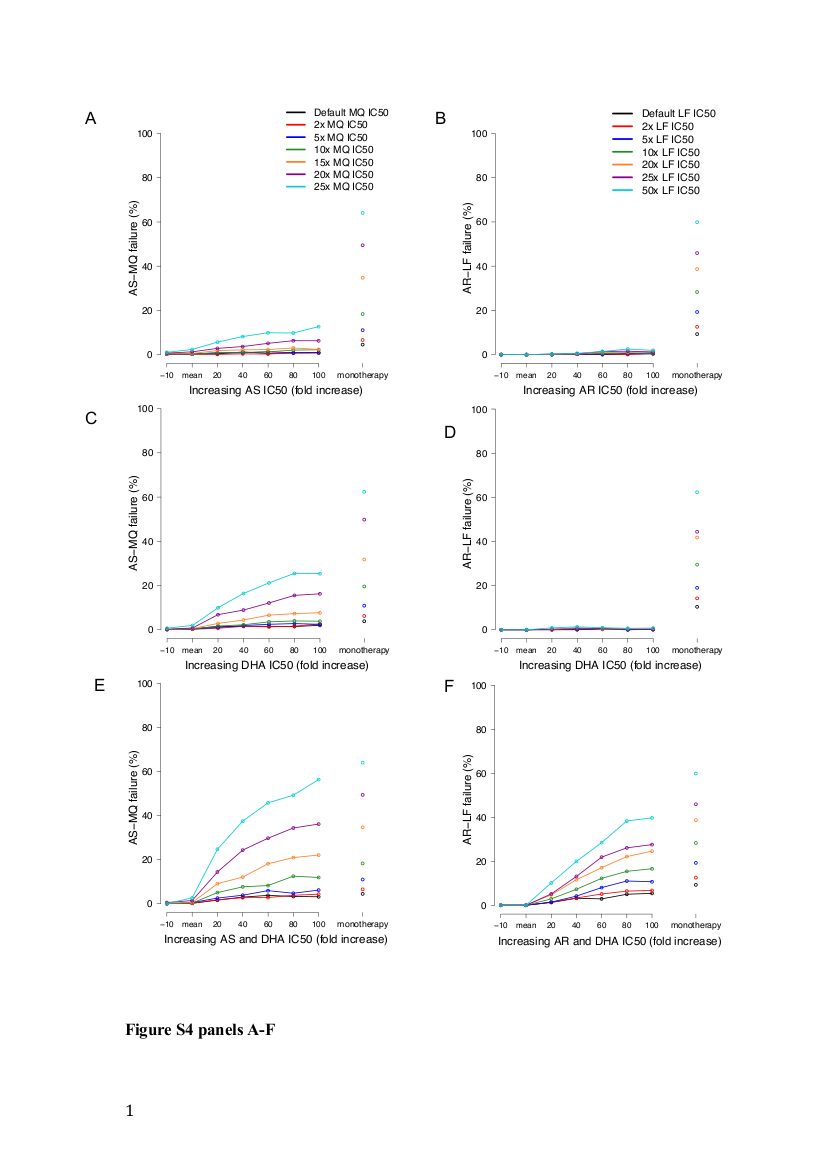

Supplement: Figure S4 — Panels A–F, changes in drug failure rates associated with increasing drug resistance when assuming independent action of the artemisinin components. Change in failure rates associated with either increasing AS/AR IC50 (panels A–B), increasing DHA IC50 (panels C–D) or increasing AS/AR and DHA IC50 (panels E–F), AS-MQ treatment (left column) and AR-LF treatment (right column) assuming independent action of the artemisinin components. Note that failure rates for monotherapies are shown as columns to the immediate right of the x-axis. (TIF) [file pcbi.1003151.s004.tif]

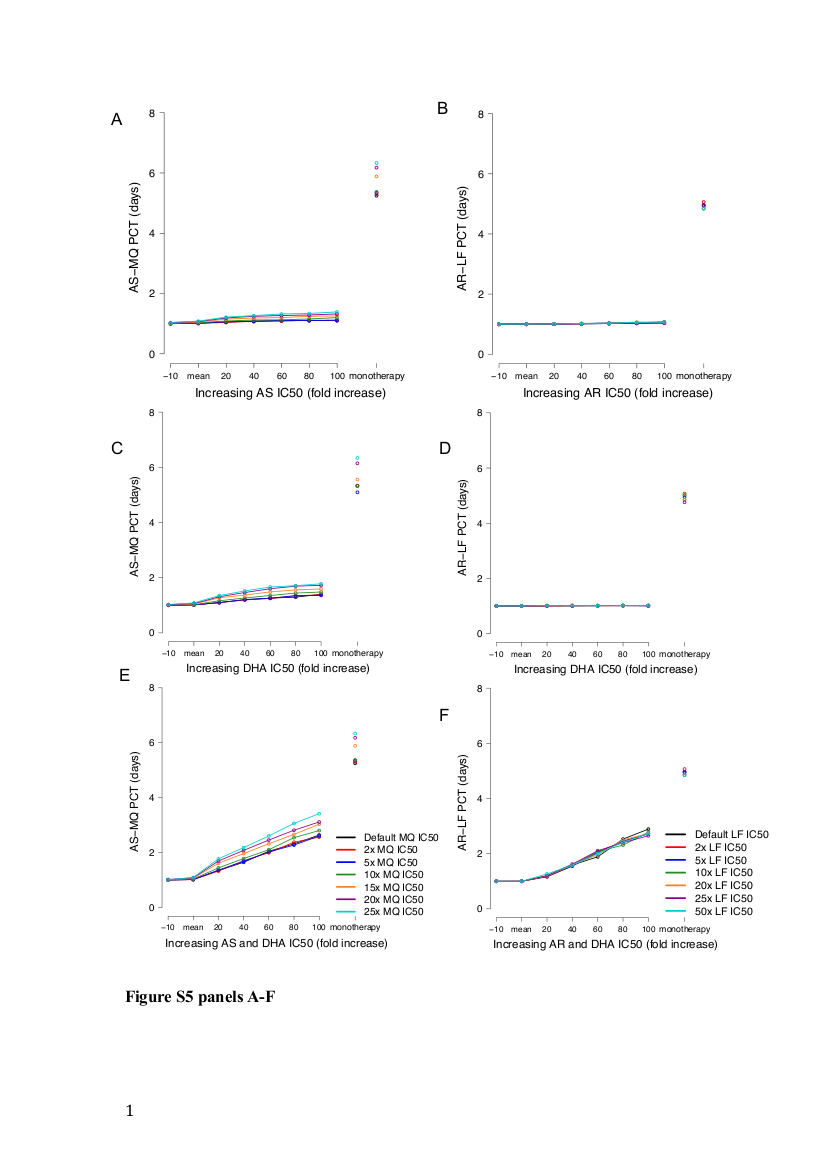

Supplement: Figure S5 — Panels A–F, changes in parasite clearance times associated with increasing drug resistance when assuming independent action of the artemisinin components. Change in parasite clearance times (PCT) associated with either increasing AS/AR IC50 (panels A–B), increasing DHA IC50 (panels C–D) or increasing AS/AR and DHA IC50 (panels E–F), AS-MQ treatment (left column) and AR-LF treatment (right column) assuming independent action of the artemisinin components. Note that PCTs for monotherapies are shown as columns to the immediate right of the x-axis. (TIF) [file pcbi.1003151.s005.tif]

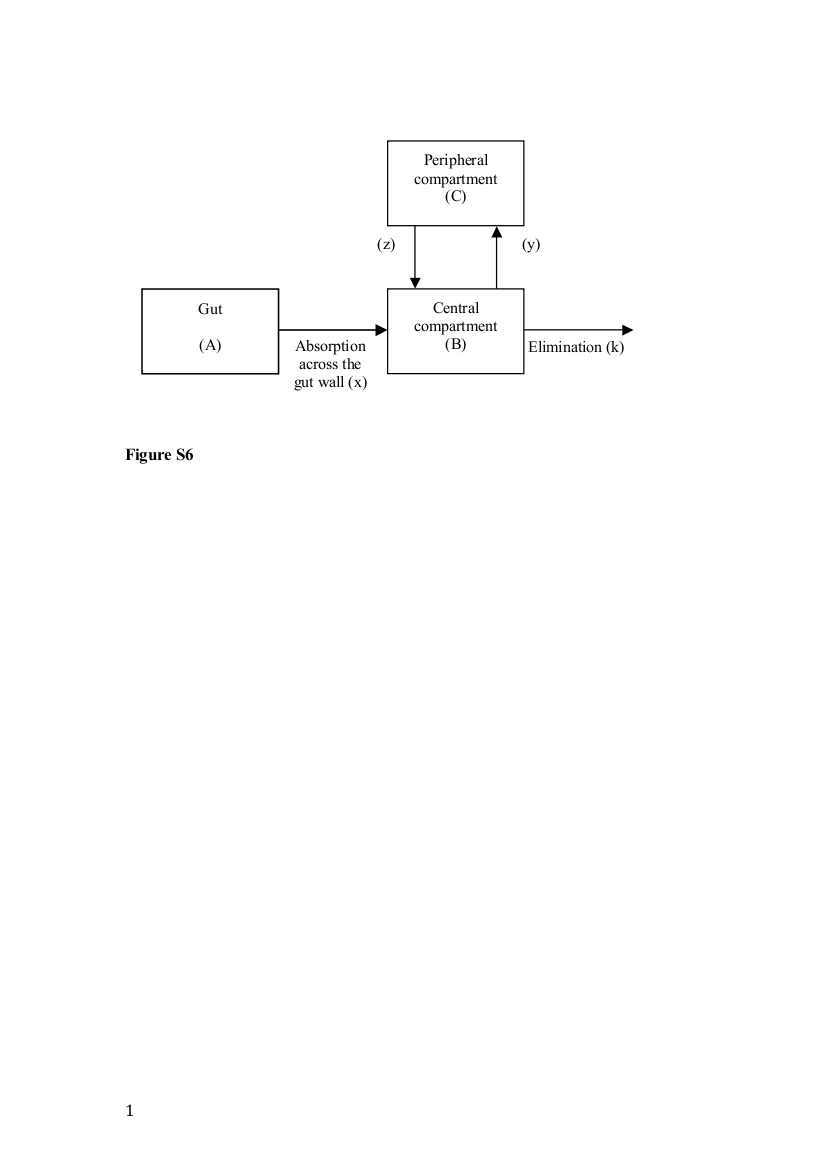

Supplement: Figure S6 — The standard two-compartment pharmacokinetic model. A standard PK two-compartment model allowing for drug absorption from the gut (component A) to the central compartment (component B) at a rate x. The drug is either eliminated from the body at a rate k or exchanged with a peripheral compartment (component C)), the drug leaves the central compartment at a rate y and returns at a rate z. (TIF) [file pcbi.1003151.s006.tif]
