# Supplementary material for: Improving Pharmacokinetic-Pharmacodynamic Modeling to Investigate Anti-Infective Chemotherapy with Application to the Current Generation of Antimalarial Drugs
Source: PLoS Comput Biol. 2013 Jul 18;9(7):e1003151. doi: 10.1371/journal.pcbi.1003151 (PMC3715401; doi:10.1371/journal.pcbi.1003151)
Supplement: Table S1 — Mean antimalarial drug parameters and their associated distributions. Mean antimalarial drug parameters for artesunate-mefloquine and artemether-lumefantrine combination therapies. The amount of variation (i.e. CV) is given in square brackets. (DOCX) [file pcbi.1003151.s007.docx]

**Table S1.**

|  | Artesunate-Mefloquine | | | Artemether-Lumefantrine | | |
| --- | --- | --- | --- | --- | --- | --- |
|  | Artesunate | DHA | Mefloquine | Artemether | DHA | Lumefantrine |
| Vd  (l/kg) | 7·1^[^[^3^](#_ENREF_3)^]^  [94^[^[^29^](#_ENREF_29)^]^] | 1·49^[^[^3^](#_ENREF_3)^]^  [48^[^[^30^](#_ENREF_30)^]^] | 20·8^[^[^31^](#_ENREF_31)^]^  [38^[^[^32^](#_ENREF_32)^,^ [^33^](#_ENREF_33)^]^] | 5·21^[^[^2^](#_ENREF_2)^]^  [82^[^[^34^](#_ENREF_34)^]^] | 3·7^[^[^2^](#_ENREF_2)^]^  [48^[^[^30^](#_ENREF_30)^]^] | 21^[^[^35^](#_ENREF_35)^]^  [263^[^[^20^](#_ENREF_20)^,^ [^36^](#_ENREF_36)^]^] |
| x  (/day) | 252^[^[^3^](#_ENREF_3)^]^  [112^[^[^37^](#_ENREF_37)^]^] | - | - | 23·98^[^[^2^](#_ENREF_2)^]^  [68^[^[^5^](#_ENREF_5)^,^ [^34^](#_ENREF_34)^]^] | - | - |
| z  (/day) | 30·96^[^[^3^](#_ENREF_3)^]^  [36·2^[^[^37^](#_ENREF_37)^]^] | - | - | 11·97^[^[^2^](#_ENREF_2)^]^  [65^[^[^5^](#_ENREF_5)^,^ [^34^](#_ENREF_34)^]^] | - | - |
| k  (/day) | - | 25·4^[^[^3^](#_ENREF_3)^]^  [23^[^[^37^](#_ENREF_37)^,^ [^38^](#_ENREF_38)^,^ [^39^](#_ENREF_39)^,^ [^40^](#_ENREF_40)^]^] | 0·053^[^[^41^](#_ENREF_41)^]^  [63^[^[^33^](#_ENREF_33)^]^] | - | 44·15^[^[^2^](#_ENREF_2)^]^  [23^[^[^37^](#_ENREF_37)^,^ [^38^](#_ENREF_38)^,^ [^39^](#_ENREF_39)^,^ [^40^](#_ENREF_40)^]^] | 0·16^[^[^15^](#_ENREF_15)^,^ [^41^](#_ENREF_41)^,^ [^42^](#_ENREF_42)^]^  [5^[^[^39^](#_ENREF_39)^]^] |
| IC50  (mg/l) | 0·0016^(^[^2^](#_ENREF_2)^,^ [^18^](#_ENREF_18)^)^  [86^[^[^30^](#_ENREF_30)^]^] | 0·009^[^[^30^](#_ENREF_30)^]^  [117^[^[^30^](#_ENREF_30)^]^] | 0·027^[^[^43^](#_ENREF_43)^]^  [78^[^[^30^](#_ENREF_30)^]^] | 0·0023^*^  [79^[^[^44^](#_ENREF_44)^]^] | 0·009^[^[^30^](#_ENREF_30)^]^  [117^[^[^30^](#_ENREF_30)^]^] | 0·032^[^[^43^](#_ENREF_43)^,^ [^45^](#_ENREF_45)^]^  [102^[^[^30^](#_ENREF_30)^]^] |
| V_max_ | 27·6^†^ | 27·6^†^ | 3·45^[^[^31^](#_ENREF_31)^]^ | 27·6^†^ | 27·6^†^ | 3·45^[^[^31^](#_ENREF_31)^]^ |
| n | 4^[^[^45^](#_ENREF_45)^]^ | 4^[^[^45^](#_ENREF_45)^]^ | 5^[^[^45^](#_ENREF_45)^]^ | 4^‡^ | 4^[^[^45^](#_ENREF_45)^]^ | 4^[^[^45^](#_ENREF_45)^]^ |

Vd: Volume of distribution; x: Absorption rate constant; z: Conversion rate; k: Elimination rate constant; IC50: Concentration producing half the desired effect; V_max_: First order rate constant of parasite killing per day; n: slope factor.

^*^ Unpublished data from Liverpool School of Tropical Medicine

^‡^Assumed to be like artesunate

^†^ As described in the Methods of the main manuscript
