# Supplementary material for: Improving Pharmacokinetic-Pharmacodynamic Modeling to Investigate Anti-Infective Chemotherapy with Application to the Current Generation of Antimalarial Drugs
Source: PLoS Comput Biol. 2013 Jul 18;9(7):e1003151. doi: 10.1371/journal.pcbi.1003151 (PMC3715401; doi:10.1371/journal.pcbi.1003151)
Supplement: Table S2 — Correlations between the IC50s of five antimalarial drugs. Data describing the half-maximal inhibitory concentration (IC50) of 5 different antimalarials measured in 7 different P.falciparum strains by Delves et al. [46], was used to determine whether the IC50s of the artemisinins are correlated. (DOCX) [file pcbi.1003151.s008.docx]

**Table S2.**

|  |  | Artemether | Artemisinin | Artemisone | DHA |
| --- | --- | --- | --- | --- | --- |
| Artemisinin | Pearson Correlation (Sig. 2-tailed) | 0·893**  p = 0.007 | 1 |  |  |
| Artemisone | Pearson Correlation (Sig. 2-tailed) | 0·439 | 0·347 | 1 |  |
| DHA | Pearson Correlation (Sig. 2-tailed) | 0·537 | 0·506 | 0·703 | 1 |
| Artesunate | Pearson Correlation (Sig. 2-tailed) | 0·717 | 0·802*  p = 0·03 | 0·762*  p = 0·047 | 0·831*  p = 0·02 |
