# Supplementary material for: Improving Pharmacokinetic-Pharmacodynamic Modeling to Investigate Anti-Infective Chemotherapy with Application to the Current Generation of Antimalarial Drugs
Source: PLoS Comput Biol. 2013 Jul 18;9(7):e1003151. doi: 10.1371/journal.pcbi.1003151 (PMC3715401; doi:10.1371/journal.pcbi.1003151)
Supplement: Text S1 — Pharmacokinetic model extensions, model calibration and implementation. Supporting information. (DOCX) [file pcbi.1003151.s009.docx]

**Text S1**

**Pharmacokinetics – incorporating the absorption, conversion and elimination of drugs**

The artemisinin model outlined in Figure 1 was described using Equations 2 to 4 in the main text. These three differential equations were used to describe the change in the amount of drug in the gut (Equation 2) and the amount of unconverted and converted drug in the serum (Equations 3 and 4 respectively). Using Laplace transforms and the convention [[1](#_ENREF_1)] of overhead bars to indicate transformed variables, we transform the equations as follows.

Equation 2, describing the amount of drug in the gut, becomes


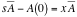
 or


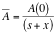


where A(0) is the amount of drug present at time zero in the gut which equals the dosage administered (*D*) plus any drug present from previous treatments (*A`*) giving


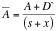
 (1.1)

Equation 1.1 can be solved by substituting *p=A`+D*


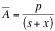


back transforming into the time domain and re-substituting gives


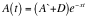
 (1.2)

Note, if no treatments are present (i.e. if *A`=0*), Equation 1.2 becomes


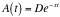
 (1.3)

Equation 3, describing the amount of unconverted drug in the serum, becomes


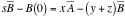
 or


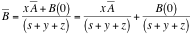


the drug is not given intravenously so *B(0)*=*B`*, the drug present from previous treatments. Substituting from Equation 1.1 gives

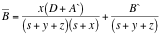
 (1.4)

Equation 1.4 can now be solved by substituting *p=x(D+A`)* and *q=(y+z)* to give


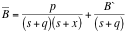


back transforming into the time domain and re-substituting gives


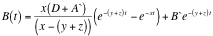
 (1.5)

Note, if no previous treatments are present (i.e. if *A`=0* and *B`=0*), *B(t)* becomes


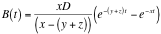
 (1.6)

Equation 4, describing the amount of converted drug in the serum, becomes


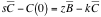
 or


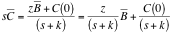


where *C(0)*=*C`*, the amount of drug present from previous treatments. Note, the fraction was split at this point to help with the transformations later. Substituting from Equation 1.6 gives

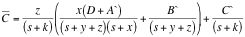
 or


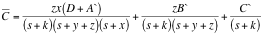
 (1.7)

Equation 1.7 can now be solved by substituting *p=zx(D+A`)*, *q=(y+z)* and *r=zB`*


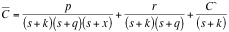


Back transforming into the time domain and re-substituting gives


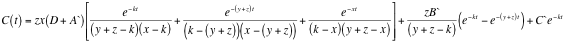


(1.8)

Tracking the amount of drug in mg requires that the changes in the molecular weight be accounted for, this was done using the ratio of the molecular weights (see main text for more information).

Note, if drug from previous treatments are absent because it is the first dose of the regimen (i.e. if *A`=0,* *B`=0* and *C`=0*), *C(t)* becomes


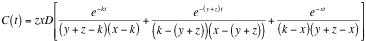
 (1.9)

The two-compartment model outlined in Figure S6 describes the change in the amount of drug in the gut (Equation 2 in main text) and the amount of drug in the central and peripheral compartments. This two-compartment model can be described using two additional differential equations


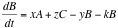
 (1.10)


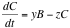
 (1.11)

Using Laplace transforms and the convention [[1](#_ENREF_1)] of overhead bars to indicate transformed variables, we transform the equations as follows.

Equation 1.10, describing the amount of drug in the central compartment, becomes


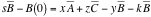
 or


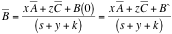
 (1.12)

*B(0)=B`* is the drug present from the previous treatments.

Equation 1.11, describing the amount of drug in the peripheral compartment, becomes


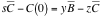
 or


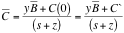
 (1.13)

where *C(0)* is the amount (mg) of drug in the peripheral compartment at the immediate end of the previous time period, denoted by C`.

Before back transforming
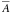
 (Equation 1.1) and
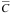
 must be substituted into Equation 1.12 and
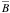
 substituted into Equation 1.13. The equations were solved in Maple 16 becoming


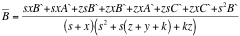
 (1.14)


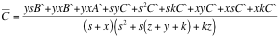
 (1.15)

The denominators of Equations 1.14 and 1.15 was simplified by factorising the quadratic equation to (s+α) (s+β), where α+β = (z+y+k) and αβ = kz, so


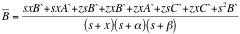
 (1.16)


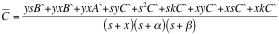
 (1.17)

The numerators of Equations 1.14 and 1.15 were simplified by substituting


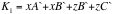


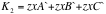


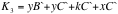


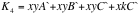


Equations 1.14 and 1.15 become


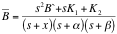
 (1.18)


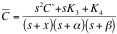
 (1.19)

These equations can now be back-transformed into the time domain using the “fingerprint” method described by Benet & Turi [[28](#_ENREF_28)], becoming


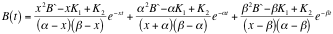
 (1.20)


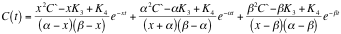
 (1.21)

**Model calibration for analysis of ACTs**

This extended model required additional model parameters to describe the absorption rate across the gut, the conversion rate to DHA and elimination of DHA following AS and AR treatments (Figure 1). All data were taken from published clinical studies, where analysis had been carried out in laboratories conforming to good laboratory practice [[2](#_ENREF_2),[3](#_ENREF_3)] i.e. patient blood samples were immediately centrifuged after sampling and the separated plasma stored at between -20 [[2](#_ENREF_2)] and -50ºC [[3](#_ENREF_3)].

Newton *et al*. [[3](#_ENREF_3)] determined the pharmacokinetic parameters of AS absorption and conversion in three adult patients in western Thailand. They used open one- and two-compartment models, fitted to plasma concentration-time data to derive standard PK parameters. Curve-fitting was performed with WinNonlin and compartment models chosen using the Akaike Information Criterion (AIC). Hietala *et al*. [[2](#_ENREF_2)] determined the pharmacokinetics of AR absorption and conversion using data from 50 paediatric patients in central Tanzania. They found the distribution of AR was best described using a two-compartment model with first-order absorption whilst DHA concentrations were best described by a covariate-free one-compartment model. The population PK/PD parameters were then determined using NONMEN version VI. Although Hietala *et al*. [[2](#_ENREF_2)] determined that a two-compartment model provided the best fit to data this was reliant on the assumption that the absorption rate constant was fixed to 1/hour.

Both studies provide estimates of the volume of distribution and elimination rate for the converted form of the drugs (DHA). The DHA estimates differed and so, for consistency, we chose to use the Newton *et al*. [[3](#_ENREF_3)] estimates when modelling AS and the Hietala *et al*. [[2](#_ENREF_2)] estimates when modelling AR (see part 4, assumptions, for further discussion). Finally, neither study showed significant routes of elimination of AS/AR from the body and so is was assumed that the drugs were fully converted to DHA (i.e. y=0).

The simulated drug concentration-time profiles of both the artemisinins using the default parameters in Table S1 are given in Figure S1 and the corresponding kill curves in Figure S2. To validate the models predictive ability the maximum serum concentration (Cmax) and time to achieve Cmax (Tmax) were compared to field data. The PK profile of AS absorption and conversion to DHA was simulated using PK parameters from Newton *et al*. [[3](#_ENREF_3)]. Figure S1.A shows both the Cmax and Tmax of AS (420ng/ml and 0.5hrs) within the range presented by Newton *et al*. [[3](#_ENREF_3)] (AS: 62-510ng/ml and 0.25-0.5hrs) and while the Cmax of DHA (600ng/ml) is slightly lower the range presented in Newton *et al*. [[3](#_ENREF_3)] (817-2853ng/ml). The Cmax range for AS is so large (see also Byakika-Kibwika *et al*.[[4](#_ENREF_4)]) that we are confident the models are consistent with clinical data. The PK profile of AR absorption and conversion to DHA was simulated using PK parameters from Hietala *et al*. [[2](#_ENREF_2)]. However, this study does not provide estimates of the corresponding Cmax and Tmax parameters and so the resulting PK profile was validated against the results of van Agtmael *et al*. [[5](#_ENREF_5)]. We note that van Agtmael *et al*. [[5](#_ENREF_5)] presents a variety of Cmax and Tmax values for both AR and DHA. For example, the drug concentration-time profiles of AR and DHA (after both AR monotherapy and AR-LF combination therapy) in Figures 1-3 of van Agtmael *et al*. [[5](#_ENREF_5)] all clearly show the Cmax of AR to be higher than DHA (although exact Cmax values vary). However, somewhat confusingly, Tables 1 and 2 (of van Agtmael *et al*. [[5](#_ENREF_5)]) show that, following AR monotherapy, the Cmax of DHA measured higher than that of AR. Whilst this observation directly contradicts the PK profiles plotted in their figures it is not discussed within the paper. For the purposes of validating the simulated PK profile, we compared the ratio of AR:DHA concentrations to those a typical patient (Figure 3, van Agtmael *et al*. [[5](#_ENREF_5)]). As in the paper, DHA Cmax (57ng/ml) was found to be approximately one third that of AR (163ng/ml). Figure S1.B also shows the simulated Tmax of AR (1·5-2 hours) and DHA (2-2·5 hours) are approximately equal to those reported in the study [[5](#_ENREF_5)].

Various studies assert that the rapid conversion of artemisinin derivatives to DHA means most of the antimalarial activity is derived from the DHA component, particularly following AS treatments (see for example [[6](#_ENREF_6),[7](#_ENREF_7),[8](#_ENREF_8),[9](#_ENREF_9),[10](#_ENREF_10)]). As a recent high profile example, Saralamba *et al*. [[7](#_ENREF_7)] state “the parasiticidal effect of artesunate was not incorporated here because the total drug exposure of artesunate was <10% of that of DHA in these patients [[11](#_ENREF_11)]”. Dondorp *et al*. [[11](#_ENREF_11)] also report DHA accounted for >90% of the artemisinin species but concentration is not directly related to killing rate (Figure S2). Antimalarial drug dosages are massive compared to IC50 levels so both entities are usually working at near-saturated killing rates. Multiplying concentration profiles in Figure S1 by their Michaelis-Menton killing (Equation 1) gives similar kill rates for both species. This is illustrated in Figure S2 where the drug kill curves for both AS/DHA and AR/DHA suggest the parent drug and the active metabolite (DHA) are both likely to contribute to the parasite killing and supported by the simulated results when the IC50’s are varied independently (see main text). While it maybe reasonable to assume that DHA is usually the dominant species it is not inevitable that this will always be the case [[11](#_ENREF_11)]. The huge amount of variation characteristic of human PK parameters (for example, see the CV estimates in Table S1) means it is inevitable that some people will slowly convert AS/AR to DHA, and rapidly eliminate the latter. Thus it is entirely plausible that AS and AR will have significant impacts in many patients and we would urge pharmacologists to measure and report their concentrations in order to understand the clinical impact.

A key operational question is whether mutations encode resistance to all artemisinins independently or whether there is any cross-resistance. Answering this question will provide crucial insights into how resistance to artemisinins is likely to spread. For example, if the IC50’s of the artemisinin forms (primarily AS, AR or DHA) are completely correlated then parasites will evolve resistance in the same way that they would to any other single drug. However if the IC50s are uncorrelated, resistance would need to be acquired to both components independently, in much the same way as it would to two drugs in combination. The latter would result in a much slower spread of resistance that the former. This can be tested if field isolates or laboratory strains are simultaneously assayed for drug sensitivity to a range of artemisinins. Unfortunately such data are rare, however the results presented here, alongside the results in a recent paper by Delves *et al*. [[12](#_ENREF_12)], have allowed us to determine the likely correlation between artemisinin IC50s. Specifically, the simulations indicate that both components of the artemisinin are active (Figure S2) while the simulated ACT failure rates and PCT only became consistent with field data when the IC50s of the artemisinins were increased simultaneously. Delves *et al*. [[12](#_ENREF_12)] describe the half maximal inhibitory concentrations (IC50s) of 39 different antimalarials measured in 7 different *P.falciparum* strains. We found the IC50 of all the artemisinin derivatives to be positively correlated (Table S2). This correlation was particularly strong (0.831; p<0.005) for AS and DHA, a drug and active metabolite routinely used as a first line treatment of malaria. We do note that a sample size of seven is small and standard deviation of each IC50 value within each isolate was often large, presumably a result of the variation in assay sensitivity. Both these factors are likely to reduce the power to detect correlations between the drugs. Despite this lack of power, all correlations were positive and 4/10 were statistically significant. Given these results and those of the simulation, it would indicate a likely correlation between the IC50 of the artemisinin components and that both the parent drug and active metabolite are responsible for the parasite killing (Figure S2) and we can conclude that the two components will be subject to joint selection pressure.

Variation was added to model parameters using parameter-specific estimates of CV.

For consistency and where possible, parameter-specific estimates of variability were taken from the same source as the default value (Table S1). Unfortunately some papers reported only the range of values measured so it was not possible to calculate a CV. In these cases, the estimates of variability were taken from other available studies (Table S1). For completeness we also include the changing failure rates seen if CV is assumed to be constant, in this case 30% (Figure S3) as in our previous study [[13](#_ENREF_13)]. It is gratifying to note that the impact of increasing levels of resistance is robust to how the CV was assigned (compare Figure S3 with Figure 2, panels E and F in the main text).

The CVs were used to determine the distribution of parameters but were often so large that a significant proportion of negative results (which are biologically impossible and hence unusable) would have occurred if we had assumed a normal distribution. We therefore assumed those parameters with a CV of <50% to be normally distributed whilst those with a CV >50% were log-normally distributed.

For log-normally distributed parameters, the logarithmic mean, *µ*, was found using


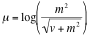
 (2.1)

where *m* was the arithmetic mean value, which in this case was equivalent to the default value (Table S1) and *v* was the variance. Here, the variance was equal to the arithmetic mean multiplied by the CV, squared.

The standard deviation, *σ*, of the log-normally distributed parameter is


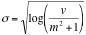
 (2.2)

The parameter value obtained from the log normal distribution was then back converted for use in the model by finding the exponential of the randomly generated number.

Regardless of the parameter distribution all random numbers generated must be positive. Each time a number was generated the program checked for values less than 0 and, if necessary, generated another random number in the same way until a positive value was chosen. Random parameter values were generated using these distributions to simulate the PK properties of individual patients and PD profiles of their infections.

The mechanistic PK/PD model presented here has met the methodological challenges involved in incorporating the absorption and conversion phases of the artemisinins whilst also tracking the concentration of more than two drugs. However, the new model structure required one further assumption, that all artemisinin species could be adequately described with a one-compartment model structure (i.e. only one compartment besides the gut is investigated, in this case the serum). There is currently considerable uncertainty in the literature as to which structural PK model provides the best fit to data for the artemisinin derivatives. Simpson *et al*. [[8](#_ENREF_8)] note that many studies of the artemisinin derivatives have either been unable to fit satisfactory PK models [[14](#_ENREF_14),[15](#_ENREF_15),[16](#_ENREF_16),[17](#_ENREF_17)], fit only a one-compartment model [[17](#_ENREF_17),[18](#_ENREF_18),[19](#_ENREF_19)] or required some PK parameters to be fixed [[16](#_ENREF_16),[17](#_ENREF_17),[18](#_ENREF_18)]. Given the confusion, we continue to assume a one-compartment model for all artemisinin species is satisfactory but the methodology could be easily extended to two-compartment models although this would require estimating and including additional PK parameters. It was also assumed that both artemisinin species have the same mode of action and so only the ‘dominant’ form with the higher kill rate was used. While this assumption is reasonable, the new methods (allowing more the action of more than two drugs simultaneously) mean it can easily be relaxed.

The extent of drug absorption and bioavailability can cause significant variability in the outcome of drug treatments. This is particularly true in the case of lumefantrine where the oral bioavailability is highly dependent on food intake and often poor in cases of acute malaria [[20](#_ENREF_20)]. When running simulations, we assumed all patients had uncomplicated malaria and followed dosing regimens precisely thus allowing us to ignore any potential complications arising from bioavailability and absorption.

The effect of combination therapies on parasite numbers were modelled assuming that partner and artemisinin act independently and that drug effect is additive, i.e. no synergy or antagonism. This was a reasonable assumption in previous simulations [[13](#_ENREF_13)] where the ACTs were modelled assuming the partner drug and only one active component for the artemisinins (i.e. instant absorption and if necessary converted) acted upon the parasites. The new methodologies described herein explicitly allow for the action of both artemisinin components (i.e. the parent drug and active metabolite) but to assume that this effect is independent and thus additive seems unrealistic given their similar modes of action. As such we chose to use only the dominant drug form (parent or metabolite) with the higher kill rate to influence the parasites over each time step. However, given the methodological extensions allowing for the action of more than two drugs simultaneously, this assumption can easily be relaxed. For completeness, the results of simulations allowing for independent action of the two artemisinin components has been included (Figure S4 and S5). We also note that this assumption that drug action is additive cannot be extended for combinations such as sulfadoxine-pyrimethamine and atovaquone-proguanil which may show synergy. Unfortunately, quantifying and even defining “synergy” or “antagonism” seems to be a topic of much debate; Chou [[21](#_ENREF_21)] discusses the “controversy and confusion” surrounding drug combinations whilst Greco *et al*. [[22](#_ENREF_22)] list no less than 13 different methods of determining synergy. With no consensus method available to define drug synergy mathematically, the best method of inclusion is likely to be the empirical approach taken for SP by Gatton *et al*. [[23](#_ENREF_23)].

When looking for the addition parameters required to describe artemisinin absorption and conversion it became apparent that the estimates of DHA volume of distribution and elimination rate differed depending on whether the metabolite was measured following treatment with AS or AR [[2](#_ENREF_2),[3](#_ENREF_3)]. It is not clear whether this response is a real biological phenomenon, for example AS and AR may differentially induce DHA elimination processes, or whether it reflects normal inter-study variability. Using two different estimates of DHA PK was obviously not ideal but with no way to choose between the estimates and for consistency with the other studies [[2](#_ENREF_2),[3](#_ENREF_3)], it seemed reasonable to use both. The need for consistency was also the reason estimates of the volume of distribution for AS and AR differed from those previously published in Winter & Hastings [[13](#_ENREF_13)].

While we do use multiple dosing regimens there was assumed to be no change in PK parameters due to auto-induction enzymes nor change due to improved clinical status after treatment has started. Running the model in shorter time steps would of course allow for these factors to be easily incorporated but were omitted here in the interests of simplicity.

**Implementation**

This model was implemented in R (version 2.9.2) [[24](#_ENREF_24)] although earlier versions were run in Maple (version 13). Both packages gave the same result but the results presented here were generated in R. Substituting Equations 7 and, where appropriate, 8 for each drug into Equation 11 enabled us to track parasite numbers and while the resulting equation was complicated, it was solved numerically using R (using the “integrate” command in the “stats” package). The model ran in half-day time steps for the first seven days to allow for multiple dosing and one-day time steps thereafter to speed up simulations. We chose to use numerical integration of half/single day time steps as it is more explicit allowing us to give dosages twice per day and, if required, change PK parameters over the course of treatment to reflect changes in the auto-induction of enzymes (as in quinine [[25](#_ENREF_25)]). However we do note that it is possible to find the treatment outcome algebraically after the final dose [[26](#_ENREF_26)].

The dosing regimens investigated were AS-MQ (4mg/kg/day AS with 8.3mg/kg/day MQ for three days) or AR-LF (1.7mg/kg AR with 12mg/kg LF given twice daily for three days) [[27](#_ENREF_27)]. The PK/PD parameter estimates are given in Table S1 and unless otherwise specified (Supporting Information, part 2), were previously validated in Winter & Hastings [[13](#_ENREF_13)].

**References**

1. Mayersohn M, Gibaldi M (1970) Mathematical methods in pharmacokinetics. Use of the laplace transformation for solving differential equations. Amer J Pharm Ed 34: 608-614.

2. Hietala SF, Martensson A, Ngasala B, Dahlstrom S, Lindegardh N, et al. (2010) Population Pharmacokinetics and Pharmacodynamics of Artemether and Lumefantrine during Combination Treatment in Children with Uncomplicated Falciparum Malaria in Tanzania. Antimicrob Agents Chemother 54: 4780-4788.

3. Newton P, Suputtamongkol Y, Teja-Isavadharm P, Pukrittayakamee S, Navaratnam V, et al. (2000) Antimalarial bioavailability and disposition of artesunate in acute falciparum malaria. Antimicrob Agents 44: 972-977.

4. Byakika-Kibwika P, Lamorde M, Okaba-Kayom V, Mayanja-Kizza H, Katabira E, et al. (2012) Lopinavir/ritonavir significantly influences pharmacokinetic exposure of artemether/lumefantrine in HIV-infected Ugandan adults. J Antimicrob Chemother.

5. vanAgtmael MA, Cheng-Qi S, Qing JX, Mull R, Boxtel CJv (1999) Multiple dose pharmacokinetics of artemether in Chinese patients with uncomplicated falciparum malaria. Int J Antimicrob Agents 12: 151-158.

6. Jamsen KM, Duffull SB, Tarning J, Lindegardh N, White NJ, et al. (2011) Optimal designs for population pharmacokinetic studies of oral artesunate in patients with uncomplicated falciparum malaria. Malar J 10: 181.

7. Saralamba S, Pan-Ngum W, Maude RJ, Lee SJ, Tarning J, et al. (2010) Intrahost modeling of artemisinin resistance in Plasmodium falciparum. Proc Natl Acad Sci 108: 397-402.

8. Simpson JA, Jamsen KM, Price RN, White NJ, Lindegardh N, et al. (2009) Towards optimal design of anti-malarial pharmacokinetic studies. Malar J 8: 189.

9. Stepniewska K, Taylor W, Sirima SB, Ouedraogo EB, Ouedraogo A, et al. (2009) Population pharmacokinetics of artesunate and amodiaquine in African children. Malar J 8: 200.

10. White NJ (1994) Clinical pharmacokinetics and pharmacodynamics of artemisinin and derivatives. Trans R Soc Trop Med Hyg 88: S41-43.

11. Dondorp AM, Nosten F, Yi P, Das D, Phyo AP, et al. (2009) Artemisinin resistance in *Plasmodium falciparum* malaria. N Engl J Med 361: 455-467.

12. Delves M, Plouffe D, Scheurer C, Meister S, Wittlin S, et al. (2012) The Activities of Current Antimalarial Drugs on the Life Cycle Stages of Plasmodium: A Comparative Study with Human and Rodent Parasites. PLoS Med 9: e1001169.

13. Winter K, Hastings IM (2011) Development, evaluation and application of an *in silico* model for antimalarial drug treatment failure. Antimicrob Agents 55: 3380-3392.

14. Karunajeewa HA, Ilett KF, Dufall K, Kemiki A, Bockarie M, et al. (2004) Disposition of Artesunate and Dihydroartemisinin after Administration of Artesunate Suppositories in Children from Papua New Guinea with Uncomplicated Malaria. Antimicrob Agents Chemother 48: 2966-2972.

15. Mcgready R, Stepniewska K, Lindegardh N, Ashley EA, La Y, et al. (2006) The pharmacokinetics of artemether and lumefantrine in pregnant women with uncomplicated falciparum malaria. Eur J Clin Pharmacol 62: 1021-1031.

16. Mithwani S, Aarons L, Kokwaro GO, Majid O, Muchohi S, et al. (2003) Population pharmacokinetics of artemether and dihydroartemisinin following single intramuscular dosing of artemether in African children with severe falciparum malaria. Br J Clin Pharmacol 57: 146-152.

17. Simpson J, Agbenyega T, Barnes K, Perri G, Folb P, et al. (2006) Population Pharmacokinetics of artesunate and dihydroartemisinin following intra-rectal dosing of artesunate in malaria patients. Plos Med 3: 2113-2123.

18. Ezzet F, Mull R, Karbwang J (1998) Population pharmacokinetics and therapeutic response of CGP 56697 (artemether+ benflumetol) in malaria patients. Br J clin Pharmac 46: 553-561.

19. Hien TT, Davis TME, Chuong LV, Ilett KF, Sinh DXT, et al. (2004) Comparative Pharmacokinetics of Intramuscular Artesunate and Artemether in Patients with Severe Falciparum Malaria. Antimicrob Agents Chemother 48: 4234-4239.

20. Ezzet F, Vugt MV, Nosten F, Looareesuwan S, White NJ (2000) Pharmacokinetics and pharmacodynamics of lumefantrine (benflumetol) in acute falciparum malaria. Antimicrob Agents 44: 697-704.

21. Chou TC (2006) Theoretical Basis, Experimental Design, and Computerized Simulation of Synergism and Antagonism in Drug Combination Studies. Pharmacological Reviews 58: 621-681.

22. Greco WR, Park HS, Rustum YM (1990) Application of a new approach for the quantitation of drug synergism to the combination of cis-diamminedichloroplatinum and 1-beta-D-arabinofuranosylcytosine. Cancer Res 50: 5318-5327.

23. Gatton ML, Martin LB, Cheng Q (2004) Evolution of Resistance to Sulfadoxine-Pyrimethamine in Plasmodium falciparum. Antimicrob Agents 48: 2116-2123.

24. R Development Core Team (2009). R: A Language and Environment for Statistical Computing.

25. The malERA Consultative Group on Drugs (2011) A Research Agenda for Malaria Eradication: Drugs PLoS Med 8: 15-23.

26. Hoshen M, Stein W, Ginsburg H (1998) Modelling the chloroquine chemotherapy of falciparum malaria: the value of spacing a split dose. Parasitology 116: 407-416.

27. World Health Organisation (2010) Guidelines for the treatment of malaria (second edition). 1-196 p.

28. Benet LZ, Turi JS (1971) Use of general partial fraction theorem for obtaining inverse laplace transforms in pharmacokinetic analysis. J Pharm Sci 60: 1593-1594.

29. Morris CA, Onyamboko MA, Capparelli E, Koch MA, Atibu J, et al. (2011) Population pharmacokinetics of artesunate and dihydroartemisinin in pregnant and non-pregnant women with malaria. Malar J 10: 114.

30. Mayxay M, Barends M, Brockman A, Jaidee A, Nair S, et al. (2007) *In vitro* antimalarial drug susceptibility and *pfcrt* mutation among fresh *Plasmodium falciparum* isolates from the Lao PDR (Laos). Am J Trop Med Hyg 76: 245-250.

31. White NJ (1992) Antimalarial pharmacokinetics and treatment regimens. Br J clin Pharmac 34: 1-10.

32. Simpson J, Watkins E, Price R, Aarons L, Kyle D, et al. (2000) Mefloquine pharmacokinetic-pharmacodynamic models: implications for dosing and resistance. Antimicrob Agents 44: 3414-3424.

33. Karbwang J, Bangchang KN, Thanavibul A, Back DJ, Bunnag D, et al. (1994) Pharmacokinetics of mefloquine alone or in combination with artesunate. Bulletin of the World Health Organization 72: 83-87.

34. Ali S, Najmi MH, Tarning J, Lindegardh N (2010) Pharmacokinetics of artemether and dihydroartemisinin in healthy Pakistani male volunteers treated with artemether-lumefantrine. Malar J 9: 275.

35. Colussi D, Parisot C, Legay F, Lefèvre G (1999) Binding of artemether and lumefantrine to plasma proteins and erythrocytes. Eur J Pharm Sci 9: 9-16.

36. Tarning J, Lindegardh N, Sandberg S, Day NJP, White NJ, et al. (2008) Pharmacokinetics and metabolism of the antimalarial piperaquine after intravenous and oral single doses to the rat. J Pharm Sci 97: 3400-3410.

37. Tan B, Naik H, Jang I-J, Yu K-S, Kirsch LE, et al. (2009) Population pharmacokinetics of artesunate and dihydroartemisinin following single- and multiple-dosing of oral artesunate in healthy subjects. Malar J 8.

38. Chinh N, Quang N, Thanh N, Dai B, Geue JP, et al. (2009) Pharmacokinetics and bioequivalence evaluation of two fixed-dose tablet formulations of dihydroartemisinin and piperaquine in Vietnamese subjects. Antimicrob Agents 53: 828-831.

39. Djimdé A, Lefèvre G (2009) Understanding the pharmacokinetics of Coartem®. Malar J 8: S4.

40. Nguyen DVH, Nguyen QP, Nguyen ND, Le TTT, Nguyen TD, et al. (2009) Pharmacokinetics and *ex vivo* pharmacodynamic antimalarial activity of dihydroartemisinin-piperaquine in patients with uncomplicated falciparum malaria in Vietnam. Antimicrob Agents 53: 3534-3537.

41. Ward S, Sevene E, Hastings I, Nosten F (2007) Antimalarial drugs and pregnancy: safety, pharmacokinetics, and pharmacovigilance. Lancet Infect Dis 7: 136-144.

42. Giao P, Vries Pd (2001) Pharmacokinetic interactions of antimalarial agents. Clin Pharmacokinet 40: 343-373.

43. Brockman A, Price RN, Van Vugt M, Heppner DG, Walsh D, et al. (2000) *Plasmodium falciparum* antimalarial drug susceptibility on the north-western border of Thailand during five years of extensive use of artesunate-mefloquine. Trans R Soc Trop Med Hyg 94: 537-544.

44. Pradines B, Rogier C, Fusai T, Tall A, Trape JF, et al. (1998) In vitro activity of artemether against African isolates (Senegal) of Plasmodium falciparum in comparison with standard antimalarial drugs. Am J Trop Med Hyg 58: 354-357.

45. Stepniewska K, White NJ (2008) Pharmacokinetic determinants of the window of selection for antimalarial drug resistance. Antimicrob Agents 52: 1589-1596.
